# Cognitive Changes following Bilateral Deep Brain Stimulation of Subthalamic Nucleus in Parkinson's Disease: A Meta-Analysis

**DOI:** 10.1155/2016/3596415

**Published:** 2016-05-23

**Authors:** Yi Xie, Xiangyu Meng, Jinsong Xiao, Jie Zhang, Junjian Zhang

**Affiliations:** ^1^Department of Neurology, Zhongnan Hospital of Wuhan University, Wuhan 430071, China; ^2^Center for Evidence-Based and Translational Medicine, Zhongnan Hospital of Wuhan University, Wuhan 430071, China; ^3^Department of Neurosurgery, Zhongnan Hospital of Wuhan University, Wuhan 430071, China

## Abstract

*Background*. Nowadays, it has been largely acknowledged that deep brain stimulation of subthalamic nucleus (STN DBS) can alleviate motor symptoms of Parkinson's disease, but its effects on cognitive function remain unclear, which are not given enough attention by many clinical doctors and researchers. To date, 3 existing meta-analyses focusing on this issue included self-control studies and have not drawn consistent conclusions. The present study is the first to compare effect sizes of primary studies that include control groups, hoping to reveal the net cognitive outcomes after STN DBS and the clinical significance.* Methods*. A structured literature search was conducted using strict criteria. Only studies with control group could be included. Data on age, duration of disease, levodopa equivalent dosage (LED), and multiple cognitive scales were collected and pooled.* Results*. Of 172 articles identified, 10 studies (including 3 randomized controlled trials and 7 nonrandomized controlled studies) were eligible for inclusion. The results suggest that STN DBS results in decreased global cognition, memory, verbal fluency, and executive function compared with control group. No significant difference is found in other cognitive domains.* Conclusions*. STN DBS seems relatively safe with respect to cognitive function, and further studies should focus on the exact mechanisms of possible verbal deterioration after surgery in the future.

## 1. Introduction

Parkinson's disease (PD), characterized by both motor and nonmotor symptoms (NMSs) is the second most common neurodegenerative disease after Alzheimer's disease [1]. The motor symptoms include resting tremor, rigidity, bradykinesia, and postural instability, while NMSs include behavioral/neuropsychiatric changes, cognitive changes, autonomic nervous system failure, sensory disorders, and sleep disturbances [2]. According to Braak, NMSs like anosmia, autonomic disturbances, and sensory disorders can precede motor symptoms by years and seriously affect quality of life since NMSs usually do not respond to dopaminergic medication as well as motor symptoms [3]. However, it should be alerted that, compared with motor symptoms, NMSs are often underrecognized and poorly managed by clinical doctors and researchers [4].

As the well-accepted method to treat PD, long-term dopaminergic medication may cause complications such as symptom fluctuations and dyskinesia. To solve this problem, deep brain stimulation of subthalamic nucleus (STN DBS) is becoming a widely performed surgical method to treat advanced PD in recent years [5]. Electrodes are implanted into the STN, delivering chronic electric stimulation with specific amplitude, pulse width, and frequency. STN DBS can not only alleviate all motor symptoms but also reduce about 50% of posttreatment dopaminergic dosage, indicating that STN may be the best target for DBS [6].

Although it is now well accepted that STN DBS can improve motor symptoms, its effect on cognitive function is less clear. In recent years, more and more studies have focused on the influence of STN DBS on cognitive function. However, the postsurgery cognitive outcomes are not consistent, which may be due to the limited sample size and different study design methods in original studies. Some found significant decline of both semantic and phonemic verbal fluency after surgery, which was potentially related to the microlesion of left-brain regions [7]. Some declared deficits in inhibitory and executive control domain for patients receiving surgery [8], while others revealed that STN DBS had minor detrimental long-term impacts on memory and frontal lobe function [9]. Up till now, 3 meta-analyses concerning this issue brought forward discrepant cognitive results, partly due to inconsistent including criteria. So we performed this quantitative meta-analysis with more strict including criteria to evaluate cognitive changes between pre- and post-STN DBS, focusing mainly on the following cognitive domains: global cognition, memory, attention, verbal fluency, reasoning, executive function, and information process, hopefully to draw an instructive conclusion and provide useful reference for clinical practice.

## 2. Methods

### 2.1. Search Strategy

A structured search of PubMed, Cochrane library, and EMbase databases for articles written in English and Chinese Knowledge Resource Integrate (CNKI), Wanfang, and VIP databases for articles written in Chinese published up to June 2015 was performed. The following keywords were used: Parkinson' disease, subthalamic nucleus, deep brain stimulation, cognition, memory, executive, verbal fluency, attention, reasoning, information process, and controlled trials. Reference lists of the selected articles were searched manually for potential additional articles.

### 2.2. Study Selection and Quality Assessment

Studies identified from primary literature search were screened for eligibility according to the following criteria.

*Inclusion Criteria*. Inclusion criteria are as follows: (1) studies including both bilateral STN DBS group and control group (control group refers to participants diagnosed as idiopathic PD and treated with dopaminergic drugs or dopamine-agonist, such as Madopar, Sinemet, pergolide, ropinirole, and pramipexole instead of STN DBS); (2) at least 10 patients in each group followed up by researchers for at least 6 months after surgery/medication; (3) pre- and posttreatment cognitive data obtained through at least one standardized instrument; (4) study results adequately reported in the form of mean and standard deviation; (5) missing data which could be calculated with previously indicated methods described in Cochrane handbook [10].

*Exclusion Criteria*. Exclusion criteria are as follows: (1) retrospective, preclinical studies, reviews, and meta-analysis not containing original data; (2) duplicate reports of identical population and same findings; (3) DBS group not evaluated in the preoperative medication-on and postoperative medication-on/stimulation-on condition.

### 2.3. Quality Assessment

The Cochrane collaboration's tool for assessing risk of bias and Methodological Index for Nonrandomized Studies (MINORS) were used for assessing the quality of randomized controlled trials (RCTs) and nonrandomized controlled studies, respectively [10, 11].

### 2.4. Data Extraction

Basic data on demographic and pretreatment parameters such as age, duration of disease, levodopa equivalent dosage (LED), Unified Parkinson's Disease Rating Scale (UPDRS) score, and Hoehn-Yahn stage were extracted from included studies, and relevant pre- and posttreatment change data concerning the STN DBS versus control comparison for the following cognitive outcomes were extracted by two reviewers independently: Mini Mental State Examination (MMSE), Mattis Dementia Rating Scale (MDRS), Digital Span Backward, paired associate learning, Rey Auditory Verbal Learning Test-total (RAVLT-total), RAVLT-delayed recall, phonemic fluency, semantic fluency, Raven's Coloured Matrices, Stroop Color Word Test, Trail Making a, and Trail Making b. If missing data were presented in an original study for certain outcomes, then data that could be used for imputation were extracted [10].

### 2.5. Statistical Analysis

The *I* ^2^ statistic and Cochrane *Q* test were used to evaluate between-study heterogeneity. An *I* ^2^ > 50% or *P* < 0.05 indicated significant heterogeneity, and a random-effects model would be used for meta-analysis; otherwise, a fixed-effects model would be used [10]. For basic demographics and pretreatment profiles, the mean difference (MD) of STN DBS versus control was used as the effect size for pooled analysis. For comparisons of pre- and posttreatment change in cognitive scales, the standardized mean difference (SMD), that is, Cohen's *d*, was used as the effect size of meta-analysis. Pooled effect size and corresponding 95% confidence interval (95% CI) were calculated. A 95% CI not covering zero indicated statistical significance of the pooled results. Sensitivity analyses were performed by changing effect models (fixed-effects model and random-effects model), and significant discrepancy indicated that the overall results were sensitive. Publication bias was detected through funnel plots and further confirmed by Begg's test. A *P* value of < 0.05 for Begg's test was considered significant statistical publication bias, and trim-and-fill method would be used to adjust the results. All analyses were performed using Review Manager 5.2 (Nordic Cochrane Centre, Copenhagen, Denmark).

## 3. Results

### 3.1. Basic Information of Included Studies

Among 172 articles screened, 10 articles met our eligibility criteria, including 3 randomized controlled studies and 7 nonrandomized controlled trials [12–21]. Totally 797 patients were included, among which 414 had been assigned to the STN DBS group and 383 to control group.

**Figure 1:**
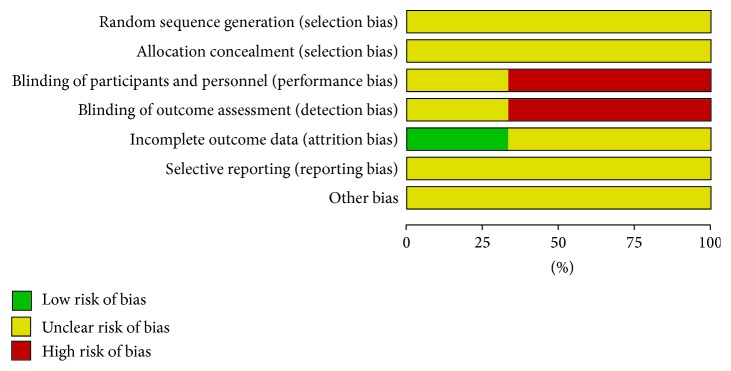
Quality assessment of RCTs using Cochrane collaboration's tool for assessing risk of bias.

Basic information (Table 1) and quality assessment results (Table 2 and Figure 1) of all the included studies were presented below.

### 3.2. Pretreatment Profile Comparison

Meta-analysis results of pretreatment profiles were shown in Table 3. According to the results, significant heterogeneity was detected for duration of disease (*I* ^2^ = 81%), LED (*I* ^2^ = 86%), and United Parkinson's Disease Rate Scale (UPDRS) in medication-on condition (*I* ^2^ = 54%). Significant difference between STN DBS and control was found for pretreatment age (MD = −1.54, 95%  CI = [−2.67, −0.41]), indicating that the STN DBS group was generally younger than the control group. Other comparisons of pretreatment profiles all yielded nonsignificant results. Forest plots of each comparison were provided in Supplementary Data (Figures S1–7) (see Supplementary Material available online at http://dx.doi.org/10.1155/2016/3596415).

### 3.3. Cognitive Change of STN DBS versus Control

Meta-analysis results of postsurgery cognitive profiles were shown in Table 4. According to the results, significant heterogeneity was detected for Stroop Color Word Test (*I* ^2^ = 75%) and Trail Making b (*I* ^2^ = 92%). Significant difference between STN DBS and control was found for the MDRS (*d* = −0.21, 95%  CI = [−0.42, −0.01]), RAVLT-total (*d* = −2.06, 95%  CI = [−4.06, −0.06]), RAVLT-delayed recall (*d* = −1.41, 95%  CI = [−2.23, −0.58]), phonemic fluency (*d* = −0.49, 95%  CI = [−0.66, −0.31]), semantic fluency (*d* = −0.39, 95%  CI = [−0.63, −0.15]), and Stroop Color Word (*d* = −0.20, 95%  CI = [−0.55, −0.15]), indicating slightly more decrease in these items in the STN DBS group as compared with the control group. Comparisons for other cognitive outcomes all yielded nonsignificant results. Forest plots of each comparison were provided in Supplementary Data (Figures S8, 10, 12, 14, 16, 18, 20, 22, 24, 26, 28, 30, and 32) and funnel plots in Supplementary Data (Figures S9, 11, 13, 15, 17, 19, 21, 23, 25, 27, 29, 31, and 33).

### 3.4. Sensitivity Analysis and Publication Bias

A sensitivity analysis was conducted by using both fixed- and random-effects models. As shown in Tables 3 and 4, no significant change was observed except for the outcome of duration of disease, LED, and Stroop Color Word analysis results. Funnel plots and Begg's test were used to evaluate publication bias. No publication bias was found except for the outcome of Trail Making b illustrated by funnel plots (Figure S27) and Begg's test (*P* = 0.009), but trim-and-fill method analysis did not reveal significant change of the summary estimate.

## 4. Discussion

There is growing interest in the cognitive changes of PD patients following bilateral STN DBS. The present meta-analysis of 10 controlled studies systematically analyzed patient characteristics and posttreatment cognitive outcomes of PD patients. The results indicate that chronic stimulation of STN could cause subtle decline in global cognition, memory, phonemic fluency, semantic fluency, and executive function.

Since the MDRS score contains memory item, verbal fluency item, and executive item, the decreased MDRS results may simply be attributed to decline in the above cognitive domains. Due to the difficulty in obtaining primary data, we cannot further analyze subscale to see if indeed decline on the MDRS is caused by decline in memory, verbal fluency, or executive function, but extra attention should be paid to this problem. One included article compared patients with 12-year mean disease duration and controls with 4.7 years mean disease duration, which might compromise the strength of decline in MDRS, RAVLT-total, RAVLT-delayed recall, and Stroop Color Word because the longer disease duration might contribute to the impairment in these cognitive domains [17]. Some studies have found more widespread effects of DBS on cognition using the reliable change index method rather than using differences in the group average (what is used in this study) [17, 18]. As such, it is possible that broader decline can be observed using more sensitive analyses methods, but data provided by included studies do not allow such analyses.

To date, 3 systematic reviews have examined cognitive changes after bilateral STN DBS [22–24]. One meta-analysis published in 2006 showed that STN DBS is relatively safe from a cognitive standpoint but causes significant, albeit small, declines in verbal learning, executive functions, and memory [22]. Another systematic analysis again ensured the safety of STN DBS with respect to cognitive effects in carefully selected participants during a follow-up of 6 months to 9 years. However, the effects of bilateral STN DBS on intelligence, memory, attention, and spatial visual function remain controversial [23]. A similar systematic analysis revealed that the probability of cognitive problem reaches as high as 41% due to the surgery procedure and that with preoperative clinically relevant cognitive deficits and affective alterations tends to further deteriorate after surgery [24]. Decrease in verbal fluency after STN DBS is progressively developed. On the other hand, executive function tends to decline in the early and later stage (<6 m and >5 years), while it stays unchanged in the intermediate stage (1-2 years) [23]. The present meta-analysis reveals the decrease of global cognition, memory, verbal fluency, and executive function in the follow-up from 6 months to 2 years after surgery, while no significant change of attention, reasoning, and information process is found.

In the existing reviews and systematic reviews, no consistent conclusion has been achieved in other cognitive domains except for the decrease of verbal fluency and executive function. However, most included studies are self-control studies, which cannot explain whether the posttreatment change derives from surgery or the natural process of the disease itself. All studies included in our meta-analysis are controlled studies, of which 3 are RCTs [12–21]. By comparing with control groups, we are able to rule out the effect of disease process, thus manifesting the net effect of STN DBS itself. Moreover, more strict inclusion and exclusion criteria were applied to avoid possible bias caused by different study designs. We included studies reporting cognitive outcomes in certain conditions (preoperative: medication-on; postoperative: medication-on, stimulation-on) because patients in the above conditions suffer least from motor symptoms, thus revealing the cognitive changes in optimal physical conditions, which makes our results more convincing. As the first meta-analysis only includes controlled studies, our studies show the exact effect of stimulation itself instead of natural process of disease.

The cognitive deficits in PD are characterized as executive defects in planning, initiation, and monitoring of goal-directed behaviors and working memory [25]. It has been widely accepted that STN DBS alleviates motor symptoms. Considering that nonmotor symptoms impose numerous burdens on patients' life, what role STN DBS plays in nonmotor symptoms is of great importance. STN DBS gives high frequency stimulation to STN nucleus, resulting in inhibition of its effects and involvement of neighboring structures, caused by mislocation of the electrode or the use of excessive voltages [26].

The exact mechanisms of cognitive changes after STN DBS still stay unclear. Sophisticated fiber connections exist between STN and cognition-related regions such as limbic and association areas, which might be influenced by chronic stimulation. STN provides the only excitatory fiber to basal ganglia, part of cognition related basal ganglia-thalamus-dorsal prefrontal cortex circuitry [27]. Electrical stimulation at the site of STN could result in changes of this circuitry as is shown in a study of 10 patients revealing intraoperative blood oxygen level dependent (BOLD) signal changes in both motor and limbic circuitry [28].

Several limitations of this meta-analysis should be considered. Only 10 studies met the eligibility criteria, with inconsistent quality, restricting the application of our conclusion. Although strict and scientific included criteria were applied, significant heterogeneity was still detected for certain comparisons. Its negative effect on the summary outcomes should not be neglected despite the use of randomized-effect model. Sensitivity analysis showed summary estimates of disease duration and LED and Stroop Color Word were unstable.

In conclusion, STN DBS is relatively safe for PD patients, although small decline in global cognitive function, memory, verbal fluency, and executive function is observed. Further studies should focus on the exact mechanisms and factors associated with memory, verbal fluency, and executive function decline in order to provide practical information to clinical doctors.

## Supplementary Material

The supplementary materials consisted of forest plots and funnel plots of meta-analysis results. Figures S1–S7 showed the difference of pretreatment age, disease duration, LED, UPDRS-III (medication-on), UPDRS-III (medication-off), Hoehn-Yahr (medication-on) and Hoehn-Yahr (medication-off) between DBS and control group. Figures S8, S10, S12, S14, S16, S18, S20, S22, S24, S26, S28, S30, S32 displayed the difference of cognitive scale score changes between DBS and control group. Publication bias of each comparison were visually examined through funnel plots of Figures S9, S11, S13, S15, S17, S19, S21, S23, S25, S27, S29, S31, S33.

## Figures and Tables

**Table 1:** Basic information of included studies (STN-DBS versus control).

Study^a^	Number of patients	Age^b^	Duration of disease^c^	Duration of follow-up	LED^d^
Rothlind, 2015 [12]	84/116	61.3 ± 8.5/62.3 ± 8.9	11.0 ± 5.0/12.8 ± 5.5	6 m	1291.5 ± 549.8/1290.1 ± 550.2
Witt, 2013 [13]	31/31	59.8 ± 7.5/58.9 ± 9.6	13.3 ± 5.5/13.1 ± 5.3	6 m	1244 ± 527/1204 ± 522
Daniels, 2010 [14]	60/63	60.2 ± 7.9/59.4 ± 7.5	13.8 ± 6.3/14.0 ± 6.1	6 m	1203 ± 535/1142 ± 463
Cilia, 2007 [15]	20/12	59.1 ± 7.4/61.2 ± 5.3	13.2 ± 3.1/15.2 ± 4.4	1 y	951 ± 465
Zangaglia, 2009 [16]	32/33	58.84 ± 7.70/62.52 ± 6.82	11.84 ± 5.07/9.97 ± 4.86	6, 12, 24 m	932.94 ± 409.86/1043.51 ± 304.87
York, 2008 [17]	23/27	59.5 ± 11.8/66.7 ± 8.7	12.0 ± 5.5/4.7 ± 4.4	7.1 m	1009.8 ± 445.3/358.9 ± 287.0
Williams, 2011 [18]	19/18	62.1 ± 10.3/66.6 ± 9.0	10.1 ± 6.24/10.1 ± 6.24	2 y	1017.6 ± 411.2/486.4 ± 293.0
Merola, 2014 [19]	19/16	60.11 ± 5.62/60.87 ± 5.81	12.94 ± 2.15/11.06 ± 2.93	6 y	1120 ± 328.79/1252.73 ± 430.02
Castelli, 2010 [20]	27/31	60.6 ± 6.7/60.2 ± 6.6	15.3 ± 5.1/15.6 ± 5.2	1 y	1046.1 ± 436.4/1046.1 ± 436.4
Smeding, 2006 [21]	99/36	57.9 ± 8.1/63.0 ± 9.1	13.7 ± 6.1/10.4 ± 4.6	6 m	899.3 ± 498.0/629.6 ± 304.9

^a^Family name of the first author and year of publication.

^b^Years, mean ± standard deviation.

^c^Month, mean ± standard deviation.

^d^Levodopa equivalent dose, mg/day, mean ± standard deviation.

**Table 2:** Risk of bias results assessed with methodological index for nonrandomized studies (MINORS).

Study	A stated aim of the study	Inclusion of consecutive patients	Prospective collection of data	Endpoint appropriate to the study aim	Unbiased evaluation of endpoints	Follow-up period appropriate to the major endpoint	Loss to follow-up not exceeding 5%	Prospective calculation of the study size	Total score
Cilia et al., 2007 [15]	2	0	2	2	0	2	2	0	10
Zangaglia et al., 2009 [16]	2	2	2	2	0	2	2	0	12
York et al., 2008 [17]	2	0	2	2	0	2	2	0	10
Williams et al., 2011 [18]	2	2	2	2	0	2	2	0	12
Merola et al., 2014 [19]	2	2	2	2	0	2	2	0	12
Castelli et al., 2010 [20]	2	2	2	2	0	2	2	0	12
Smeding et al., 2006 [21]	2	0	2	2	0	2	2	0	10

**Table 3:** Meta-analysis results of pretreatment parameters for STN DBS versus control.

Item	*I* ^2^ statistic	Mean and 95% CI (fixed-effect model)	Mean and 95% CI (randomized-effect model)
Age	43%	−1.54 (−2.67, −0.41)	−1.77 (−3.33, −0.21)
Duration of disease (month)	81%	0.9 (0.2, 1.59)	1.23 (−0.43, 2.89)
LED (mg/day)	86%	138.5 (75.83, 202.07)	142.9 (−27.17, 312.97)
UPDRS off-med	0	2.06 (−0.12, 4.25)	2.06 (−0.12, 4.25)
UPDRS on-med	54%	−0.89 (−2.46, 0.69)	−1.01 (−3.36, 1.34)
Hoehn-Yahr off-med	0	−0.04 (−0.19, 1.11)	−0.04 (−0.19, 1.11)
Hoehn-Yahr on-med	32%	−0.08 (−0.17, 0.02)	−0.07 (−0.19, 0.05)

CI: confidence interval; LED: levodopa equivalent dose; UPDRS: unified Parkinson disease rating scale.

**Table 4:** Meta-analysis results, sensitivity analysis, and publication bias of cognitive outcome.

Effect size	*I* ^2^ statistic	Mean ± sd (fixed-effect model)	Mean ± sd (randomized-effect model)	Bgger's test	
				*z*-value	*P*
MMSE	0	0.06 (−0.23, 0.36)	0.06 (−0.23, 0.36)	0.34	0.734
MDRS	0	−0.21 (−0.42, −0.01)	−0.21 (−0.42, −0.01)	1.22	0.221
Digit span backward	0	−0.14 (−0.33, 0.05)	−0.14 (−0.33, 0.05)	−0.34	1.000
Pair associate learning	0	−0.69 (−2.01, 0.63)	−0.69 (−2.01, 0.63)	—	—
RAVLT-total	0	−2.06 (−4.06, −0.06)	−2.06 (−4.06, −0.06)	−0.29	0.801
RAVLT-delayed recall	0	−1.41 (−2.23, −0.58)	−1.41 (−2.23, −0.58)	−2.07	0.287
Phonemic fluency	6%	−0.49 (−0.66, −0.31)	−0.48 (−0.67, −0.30)	0.75	0.452
Semantic fluency	0	−0.39 (−0.63, −0.15)	−0.39 (−0.63, −0.15)	1.02	0.308
Stroop Color Word	75%	−0.23 (−0.39, −0.06)	−0.20 (−0.55, 0.15)	1.13	0.260
Boston naming	0	0.02 (−0.21, 0.26)	0.02 (−0.21, 0.26)	1.04	0.296
Raven's color matrices	0	−0.15 (−0.56, 0.25)	−0.15 (−0.56, 0.25)	0.00	1.000
Trail Making a	0	0.03 (−0.17, 0.22)	0.03 (−0.17, 0.22)	0.34	0.734
Trail Making b	92%	0.08 (−0.10, 0.27)	−0.39 (−1.15, 0.38)	2.63	0.009
