# Supplementary material for: Cognitive Changes following Bilateral Deep Brain Stimulation of Subthalamic Nucleus in Parkinson's Disease: A Meta-Analysis
Source: Biomed Res Int. 2016 May 23;2016:3596415. doi: 10.1155/2016/3596415 (PMC4893566; doi:10.1155/2016/3596415)
Supplement: Supplementary file 1 — The supplementary materials consisted of forest plots and funnel plots of meta-analysis results. Figures S1–S7 showed the difference of pretreatment age, disease duration, LED, UPDRS-III (medication-on), UPDRS-III (medication-off), Hoehn-Yahr (medication-on) and Hoehn-Yahr (medication-off) between DBS and control group. Figures S8, S10, S12, S14, S16, S18, S20, S22, S24, S26, S28, S30, S32 displayed the difference of cognitive scale score changes between DBS and control group. Publication bias of each comparison were visually examined through funnel plots of Figures S9, S11, S13, S15, S17, S19, S21, S23, S25, S27, S29, S31, S33. [file 3596415.f1.pdf]

Study or Subgroup	Experimental Mean	Experimental SD	Experimental Total	Control Mean	Control SD	Control Total	Weight	Mean Difference IV, Random, 95% CI
Witt	59.8	7.5	31	58.9	9.6	31	8.6%	0.90 [−3.39, 5.19]
Daniels	60.2	7.9	60	59.4	7.5	63	14.0%	0.80 [−1.93, 3.53]
Castelli	60.6	6.7	27	60.2	6.6	31	11.2%	0.40 [−3.03, 3.83]
Merola	60.11	5.62	19	60.87	5.81	16	10.0%	−0.76 [−4.57, 3.05]
Rothlind	61.3	8.5	84	62.3	8.9	116	15.3%	−1.00 [−3.43, 1.43]
Cilia	59.1	7.4	20	61.2	5.3	12	8.3%	−2.10 [−6.52, 2.32]
Zangaglia	58.84	7.7	32	62.52	6.82	33	10.8%	−3.68 [−7.22, −0.14]
Williams	62.1	10.3	19	66.6	9	18	5.0%	−4.50 [−10.72, 1.72]
Smeding	57.9	8.1	99	63	9.1	36	11.4%	−5.10 [−8.47, −1.73]
York	59.5	11.8	23	66.7	8.7	27	5.5%	−7.20 [−13.03, −1.37]
**Total (95% CI)**			**414**			**383**	**100.0%**	**−1.77 [−3.33, −0.21]**

Heterogeneity: $Tau^2 = 2.62$; $Chi^2 = 15.88$, df = 9 (P = 0.07); $I^2 = 43\%$
Test for overall effect: Z = 2.22 (P = 0.03)

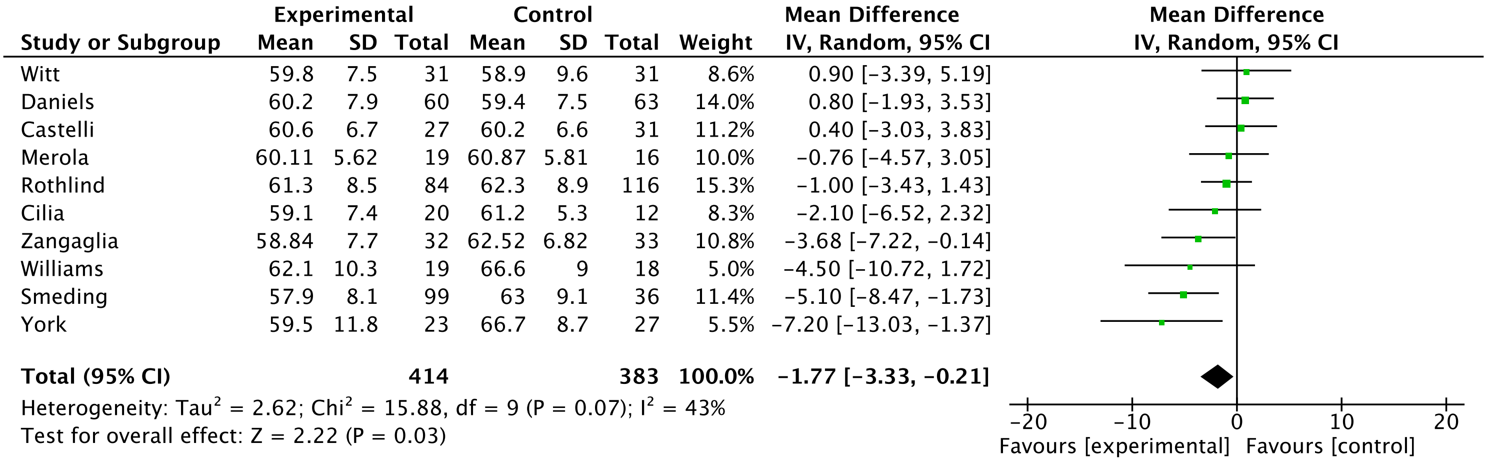

Figure S1. Meta-analysis of pretreatment age difference STN DBS vs. Control

Study or Subgroup	Experimental Mean	Experimental SD	Experimental Total	Control Mean	Control SD	Control Total	Weight	Mean Difference IV, Random, 95% CI
Castelli	15.3	5.1	27	15.6	5.2	31	9.6%	−0.30 [−2.96, 2.36]
Cilia	13.2	3.1	20	15.2	4.4	12	9.3%	−2.00 [−4.84, 0.84]
Daniels	13.8	6.3	60	14	6.1	63	10.4%	−0.20 [−2.39, 1.99]
Merola	12.94	2.15	19	11.06	2.93	16	11.2%	1.88 [0.15, 3.61]
Rothlind	11	5	84	12.8	5.5	116	11.6%	−1.80 [−3.26, −0.34]
Smeding	13.7	6.1	99	10.4	4.6	36	10.9%	3.30 [1.38, 5.22]
Williams	10.1	6.24	19	7.5	4.22	18	8.3%	2.60 [−0.82, 6.02]
Witt	13.3	5.5	31	13.1	5.3	31	9.5%	0.20 [−2.49, 2.89]
York	12	5.5	23	4.7	4.4	27	9.3%	7.30 [4.51, 10.09]
Zangaglia	11.84	5.07	32	9.97	4.86	33	10.0%	1.87 [−0.55, 4.29]
**Total (95% CI)**			**414**			**383**	**100.0%**	**1.23 [−0.43, 2.89]**

Heterogeneity: $Tau^2 = 5.64$; $Chi^2 = 48.02$, df = 9 ($P < 0.00001$); $I^2 = 81\%$
Test for overall effect: Z = 1.45 (P = 0.15)

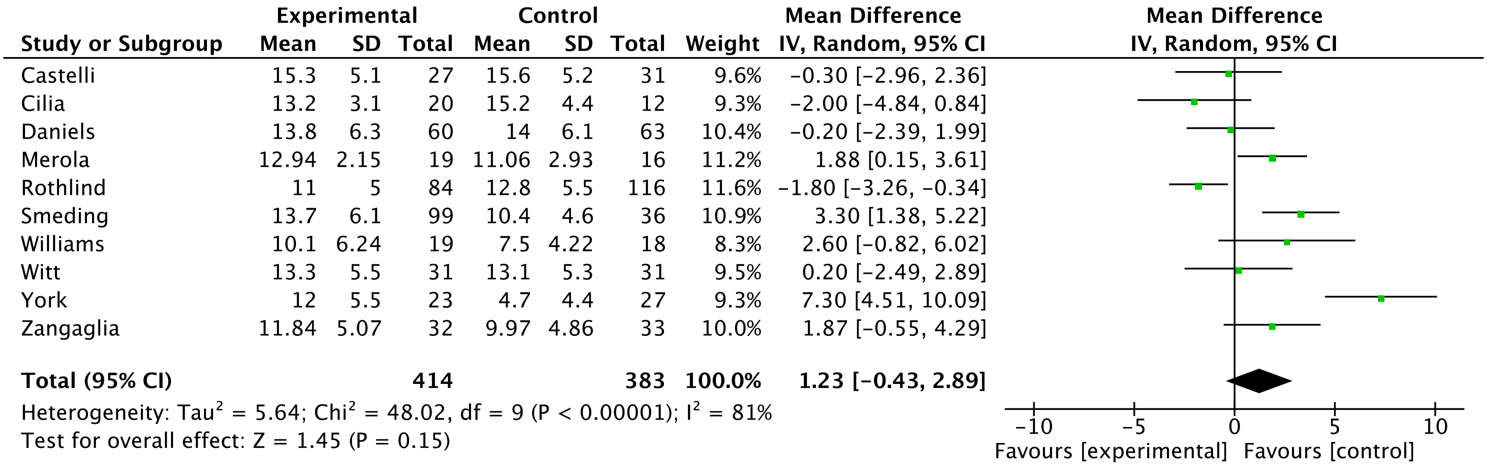

Figure S2. Meta-analysis of pretreatment duration of disease difference STN DBS vs. Control

	Experimental			Control				Mean Difference
**Study or Subgroup**	**Mean**	**SD**	**Total**	**Mean**	**SD**	**Total**	**Weight**	**IV, Random, 95% CI**
Castelli	1,046.1	436.4	27	1,071.3	370.3	31	11.0%	−25.20 [−235.17, 184.77]
Daniels	1,203	535	60	1,142	463	63	11.6%	61.00 [−116.19, 238.19]
Merola	1,120	328.79	19	1,252.73	430.02	16	10.1%	−132.73 [−390.13, 124.67]
Rothlind	1,291.5	549.8	84	1,290.1	550.2	116	11.9%	1.40 [−153.03, 155.83]
Smeding	899.3	498	99	629.6	304.9	36	12.1%	269.70 [129.90, 409.50]
Williams	1,017.6	411.2	19	486.4	293	18	10.7%	531.20 [302.05, 760.35]
Witt	1,244	527	31	1,204	522	31	10.1%	40.00 [−221.12, 301.12]
York	1,009.8	445.3	23	358.9	287	27	11.0%	650.90 [439.15, 862.65]
Zangaglia	932.94	409.86	32	1,043.51	304.87	33	11.6%	−110.57 [−286.60, 65.46]
**Total (95% CI)**			**394**			**371**	**100.0%**	**142.90 [−27.17, 312.97]**

Heterogeneity: $Tau^2 = 56993.03$; $Chi^2 = 55.76$, $df = 8$ ($P < 0.00001$); $I^2 = 86\%$
Test for overall effect: $Z = 1.65$ ($P = 0.10$)

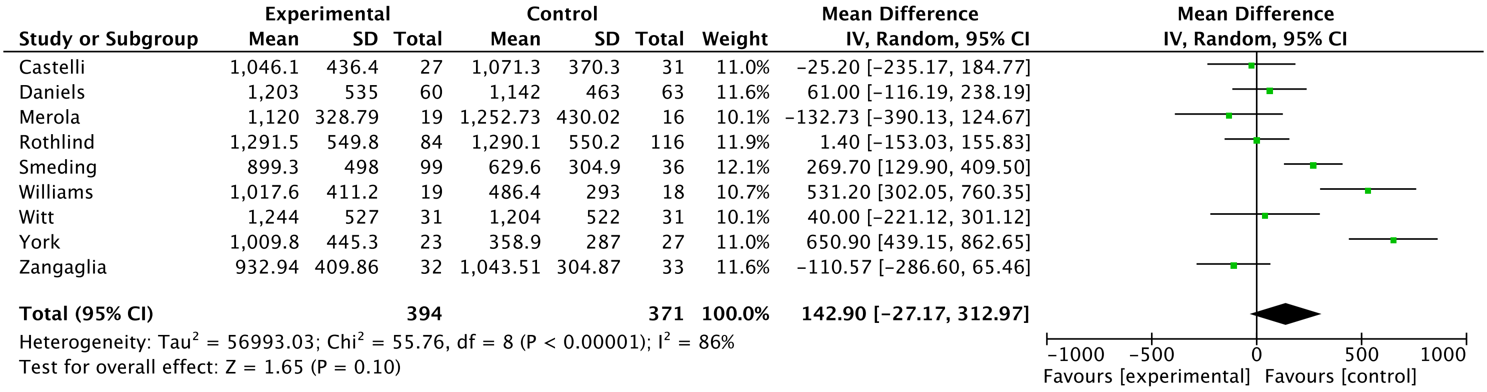

Figure S3. Meta-analysis of pretreatment LED difference STN DBS vs. Control

Study or Subgroup	Experimental Mean	Experimental SD	Experimental Total	Control Mean	Control SD	Control Total	Weight	Mean Difference IV, Random, 95% CI
Castelli	55	11.3	27	49.6	11.9	31	13.4%	5.40 [−0.58, 11.38]
Daniels	47.9	13.13	60	47.3	11.92	63	24.2%	0.60 [−3.84, 5.04]
Merola	44.37	8.09	19	40.16	10.92	16	11.4%	4.21 [−2.26, 10.68]
Rothlind	42.8	16.7	84	43.1	11.2	116	28.2%	−0.30 [−4.41, 3.81]
Witt	47.2	12.3	31	45	12.6	31	12.4%	2.20 [−4.00, 8.40]
Zangaglia	40.06	15.53	32	34.97	12.15	33	10.3%	5.09 [−1.70, 11.88]
**Total (95% CI)**			**253**			**290**	**100.0%**	**2.06 [−0.12, 4.25]**

Heterogeneity: $Tau^2 = 0.00$; $Chi^2 = 4.07$, df = 5 (P = 0.54); $I^2 = 0\%$

Test for overall effect: Z = 1.85 (P = 0.06)

Mean Difference
IV, Random, 95% CI

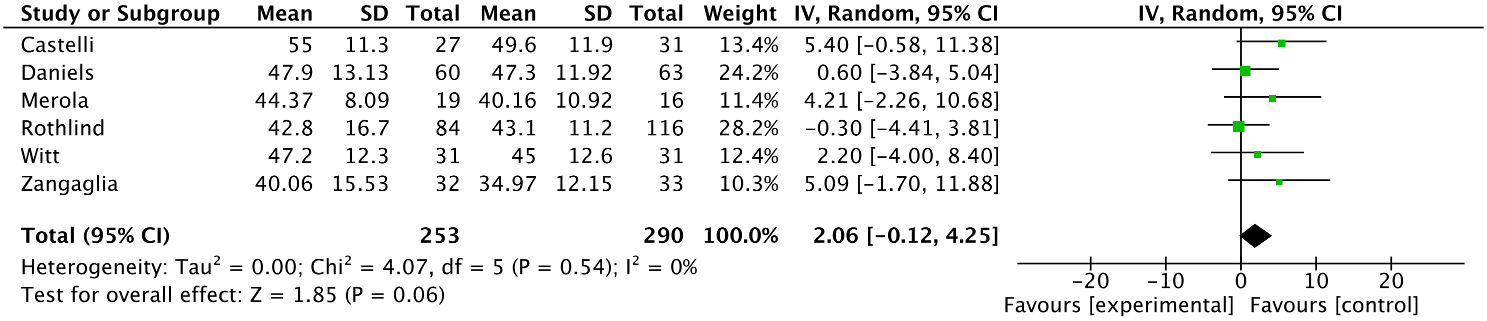

Figure S4. Meta-analysis of pretreatment UPDRS(medication-off) STN DBS vs. Control

	Experimental			Control				Mean Difference
Study or Subgroup	Mean	SD	Total	Mean	SD	Total	Weight	IV, Random, 95% CI
Daniels	18.7	9.7	60	17.3	9	63	19.4%	1.40 [−1.91, 4.71]
Merola	14.79	4.4	19	19.31	8.12	16	14.9%	−4.52 [−8.96, −0.08]
Rothlind	21.2	11.8	84	23.2	10.5	116	20.1%	−2.00 [−5.17, 1.17]
Smeding	21.2	9.2	99	25.4	13.8	36	13.5%	−4.20 [−9.06, 0.66]
Witt	19.4	8.6	31	15.9	8.5	31	15.5%	3.50 [−0.76, 7.76]
Zangaglia	18.03	8.34	32	19.16	8	33	16.6%	−1.13 [−5.10, 2.84]
**Total (95% CI)**			**325**			**295**	**100.0%**	**−1.01 [−3.36, 1.34]**

Heterogeneity: $Tau^2 = 4.56$; $Chi^2 = 10.76$, df = 5 (P = 0.06); $I^2 = 54\%$
Test for overall effect: Z = 0.84 (P = 0.40)

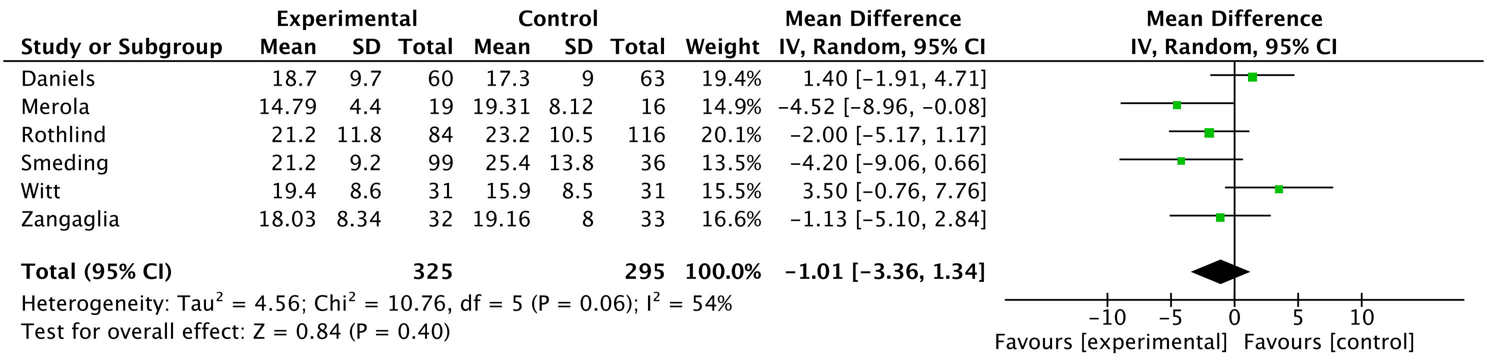

Figure S5. Meta-analysis of pretreatment UPDRS(medication-on) STN DBS vs. Control

Study or Subgroup	Experimental Mean	Experimental SD	Experimental Total	Control Mean	Control SD	Control Total	Weight	Mean Difference IV, Random, 95% CI
Daniels	3.62	0.85	60	3.77	0.86	63	25.3%	−0.15 [−0.45, 0.15]
Rothlind	3.4	0.9	84	3.3	0.9	116	36.1%	0.10 [−0.15, 0.35]
Witt	3.8	0.85	31	3.8	0.86	31	12.7%	0.00 [−0.43, 0.43]
Zangaglia	3.2	0.67	32	3.35	0.55	33	25.9%	−0.15 [−0.45, 0.15]
**Total (95% CI)**			**207**			**243**	**100.0%**	**−0.04 [−0.19, 0.11]**

Heterogeneity: $Tau^2 = 0.00$; $Chi^2 = 2.24$, df = 3 (P = 0.52); $I^2 = 0\%$
Test for overall effect: Z = 0.52 (P = 0.60)

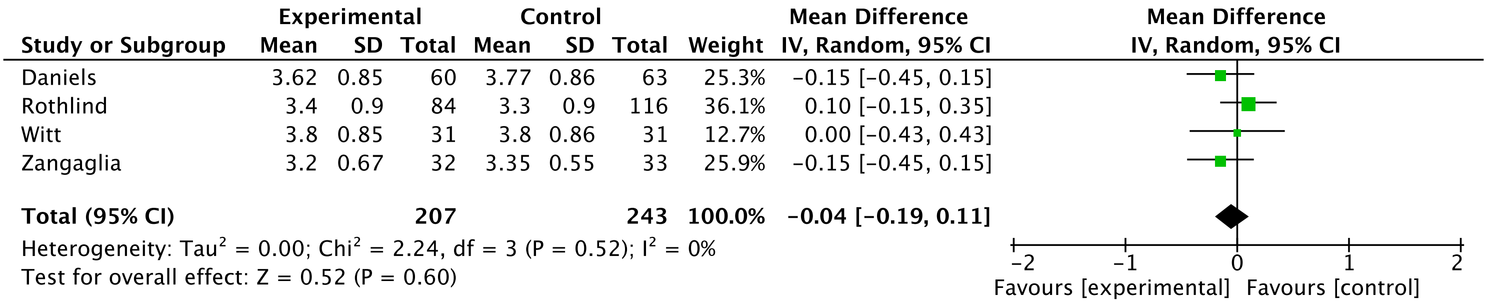

Figure S6. Meta-analysis of pretreatment Hoehn-Yahr(medication-off) STN DBS vs. Control

Study or Subgroup	Experimental Mean	Experimental SD	Experimental Total	Control Mean	Control SD	Control Total	Weight	Mean Difference IV, Random, 95% CI
Daniels	2.29	0.72	60	2.3	0.72	63	15.7%	−0.01 [−0.26, 0.24]
Rothlind	2.3	0.6	84	2.4	0.5	116	27.8%	−0.10 [−0.26, 0.06]
Smeding	2.7	0.6	99	2.7	0.7	36	15.5%	0.00 [−0.26, 0.26]
Witt	2.3	0.87	31	2.3	0.72	31	7.8%	0.00 [−0.40, 0.40]
York	2.28	0.42	23	2.13	0.58	27	13.9%	0.15 [−0.13, 0.43]
Zangaglia	2.34	0.43	32	2.64	0.47	33	19.3%	−0.30 [−0.52, −0.08]
**Total (95% CI)**			**329**			**306**	**100.0%**	**−0.07 [−0.19, 0.05]**

Heterogeneity: $Tau^2 = 0.01$; $Chi^2 = 7.38$, df = 5 (P = 0.19); $I^2 = 32\%$
Test for overall effect: Z = 1.08 (P = 0.28)

Mean Difference
IV, Random, 95% CI

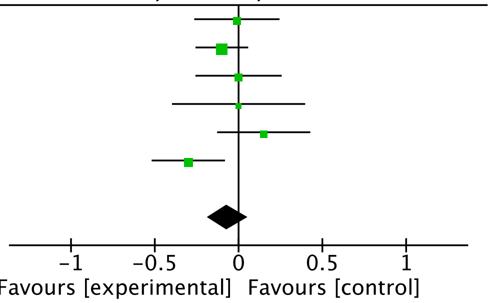

Figure S7. Meta-analysis of pretreatment Hoehn-Yahr(medication-on) STN DBS vs. Control

	Experimental			Control				Std. Mean Difference
**Study or Subgroup**	**Mean**	**SD**	**Total**	**Mean**	**SD**	**Total**	**Weight**	**IV, Fixed, 95% CI**
Cilia	0.39	1.427669	20	0.57	2.341483	12	16.5%	−0.10 [−0.81, 0.62]
Williams	−0.4	3.22259644	19	−1	1.90864159	18	20.3%	0.22 [−0.43, 0.87]
York	−0.2	3.279609	23	−0.1	2.530285	27	27.4%	−0.03 [−0.59, 0.52]
Zangaglia	−1	2.773629	32	−1.45	4.089292	33	35.8%	0.13 [−0.36, 0.61]
**Total (95% CI)**			**94**			**90**	**100.0%**	**0.06 [−0.23, 0.36]**

Heterogeneity: $Chi^2 = 0.60$, df = 3 (P = 0.90); $I^2 = 0\%$
Test for overall effect: Z = 0.44 (P = 0.66)

Std. Mean Difference
IV, Fixed, 95% CI

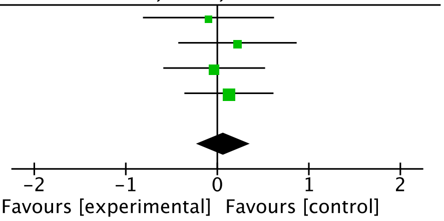

Figure S8. Forest plot of Mini Mental State Examination change STN DBS vs. Control

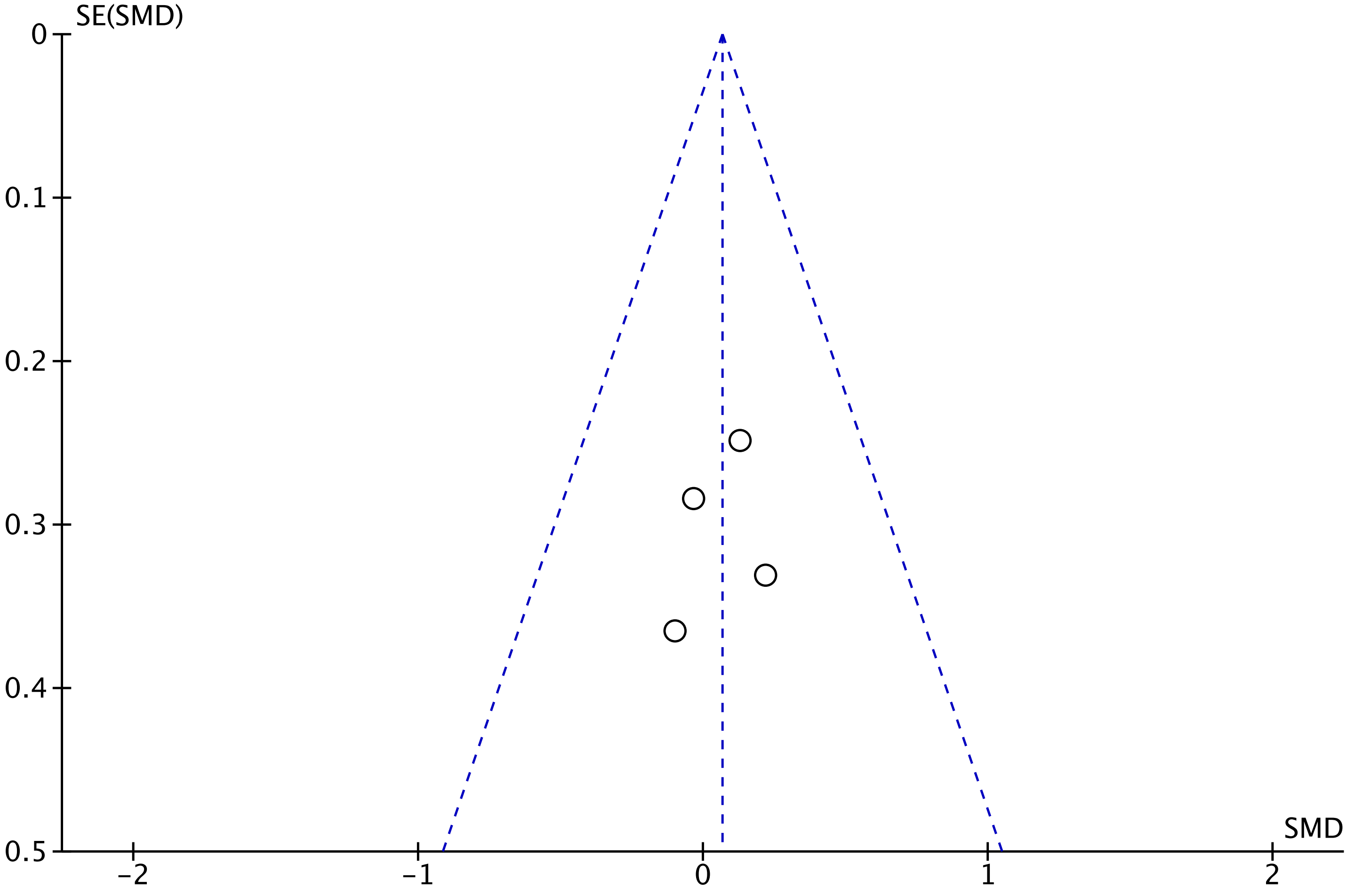

Figure S9. funnel plot of Mini Mental State Examination change STN DBS vs. Control

	Experimental			Control				Std. Mean Difference
Study or Subgroup	Mean	SD	Total	Mean	SD	Total	Weight	IV, Fixed, 95% CI
Daniels	−1.8	4.8	60	−0.7	4	63	32.6%	−0.25 [−0.60, 0.11]
Smeding	−2.3	6.8	99	−0.4	4.6	36	28.0%	−0.30 [−0.68, 0.08]
Williams	−1.5	9.67767265	19	−3.2	8.87454693	18	9.8%	0.18 [−0.47, 0.83]
Witt	−2.5	4.9	31	−1.1	4.2	31	16.4%	−0.30 [−0.80, 0.20]
York	−0.3	9.22433	23	0.6	5.48598351	27	13.2%	−0.12 [−0.68, 0.44]
**Total (95% CI)**			**232**			**175**	**100.0%**	**−0.21 [−0.42, −0.01]**

Heterogeneity: $Chi^2 = 1.88$, df = 4 (P = 0.76); $I^2 = 0\%$

Test for overall effect: Z = 2.06 (P = 0.04)

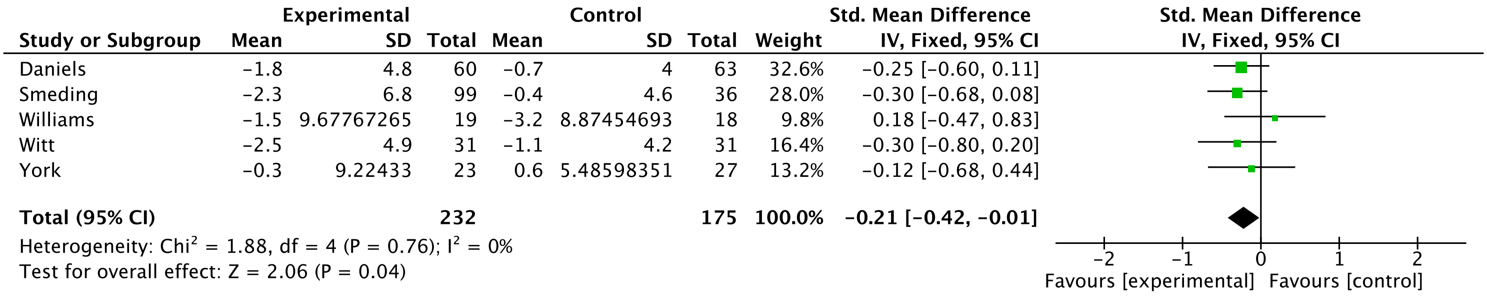

Figure S10. Forest plot of Mattis Dementia Rating Scale change STN DBS vs. Control

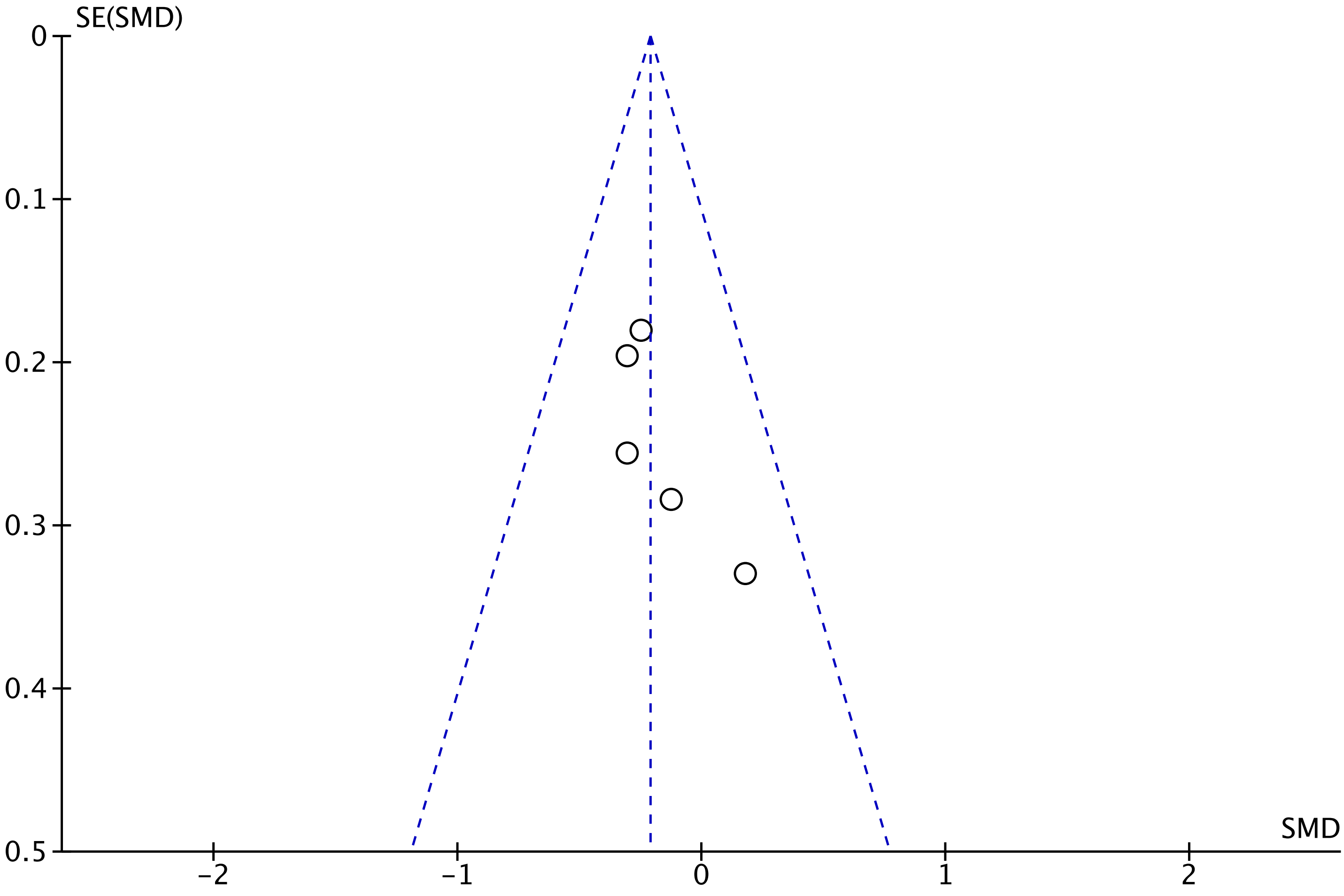

Figure S11. Funnel plot of Mattis Dementia Rating Scale change STN DBS vs. Control

	Experimental			Control				Std. Mean Difference	Std. Mean Difference
**Study or Subgroup**	**Mean**	**SD**	**Total**	**Mean**	**SD**	**Total**	**Weight**	**IV, Fixed, 95% CI**	**IV, Fixed, 95% CI**
Daniels	−0.2	1.6	60	0.1	1.9	63	28.6%	−0.17 [−0.52, 0.18]	
Rothlind	−0.1	1.7	84	0.1	1.6	116	45.5%	−0.12 [−0.40, 0.16]	
Witt	−0.6	1.6	31	0.03	1.9	31	14.3%	−0.35 [−0.86, 0.15]	
York	−0.8	3.94750547	23	−1.2	3.93372238	27	11.6%	0.10 [−0.46, 0.66]	
**Total (95% CI)**			**198**			**237**	**100.0%**	**−0.14 [−0.33, 0.05]**	

Heterogeneity: $Chi^2 = 1.46$, df = 3 (P = 0.69); $I^2 = 0\%$
Test for overall effect: Z = 1.47 (P = 0.14)

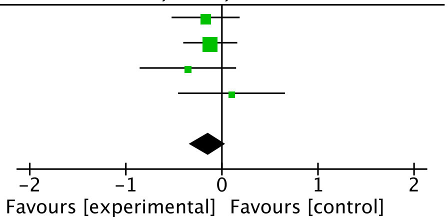

Figure S12. Forest plot of Digit span backward change STN DBS vs. Control

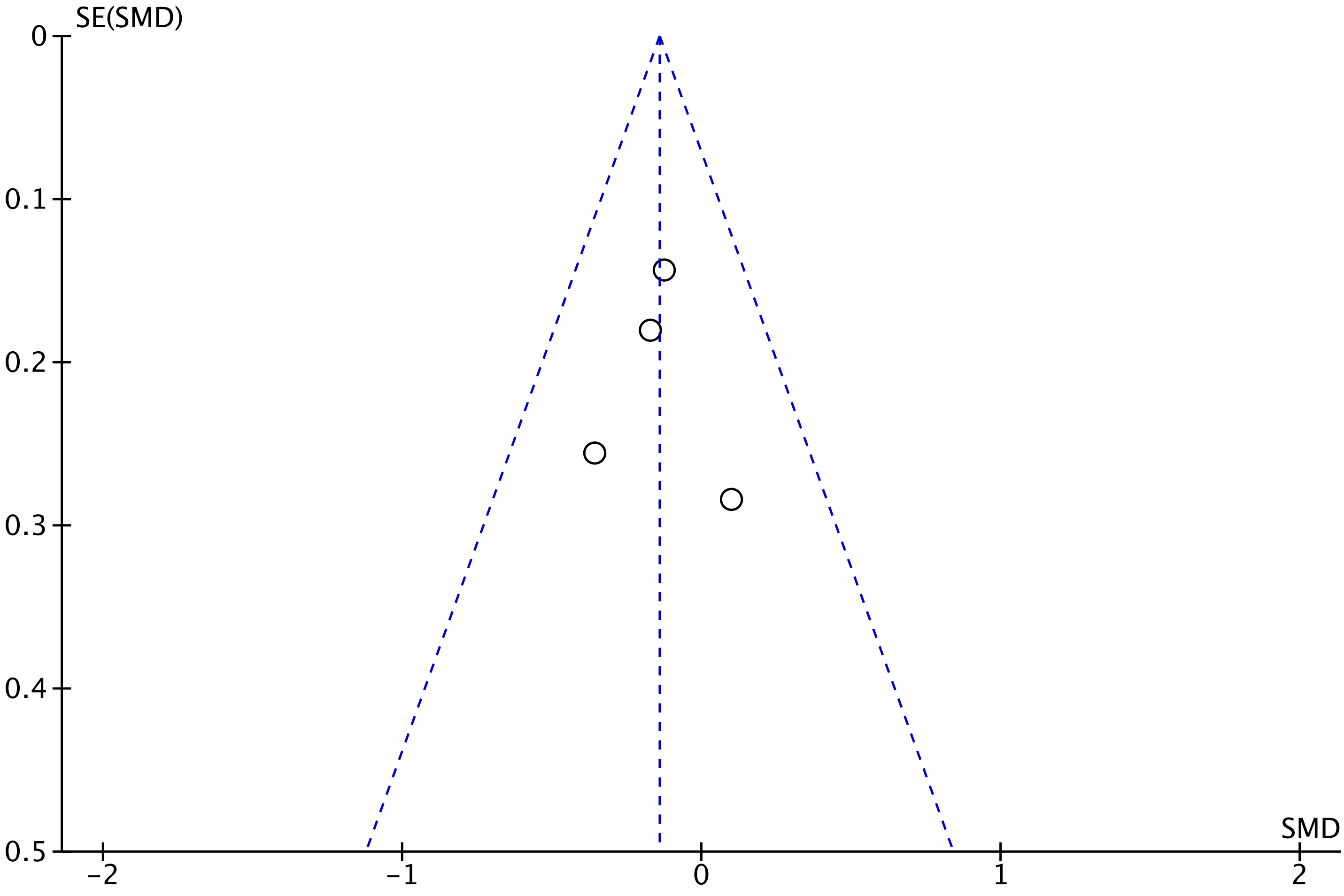

Figure S13. Funnel plot of Digit span backward change STN DBS vs. Control

Study or Subgroup	Experimental Mean	Experimental SD	Experimental Total	Control Mean	Control SD	Control Total	Weight	Std. Mean Difference IV, Fixed, 95% CI
Castelli	−5.22	12.56	27	5.31	12.13	31	10.9%	−0.84 [−1.38, −0.30]
Cilia	−3.04	10.123452	20	−3.2	9.91035786	12	6.2%	0.02 [−0.70, 0.73]
Daniels	−3.3	7	60	−0.2	6.6	63	24.8%	−0.45 [−0.81, −0.09]
Merola	−19.8	16.39966865	19	−9	17.84961071	16	6.8%	−0.62 [−1.30, 0.06]
Rothlind	−4.6	9.7	83	0.5	8.6	114	38.4%	−0.56 [−0.85, −0.27]
Witt	−1.9	8.1	31	−0.5	6	31	12.8%	−0.19 [−0.69, 0.31]
**Total (95% CI)**			**240**			**267**	**100.0%**	**−0.49 [−0.66, −0.31]**

Heterogeneity: $Chi^2 = 5.30$, df = 5 (P = 0.38); $I^2 = 6\%$
Test for overall effect: Z = 5.33 (P < 0.00001)

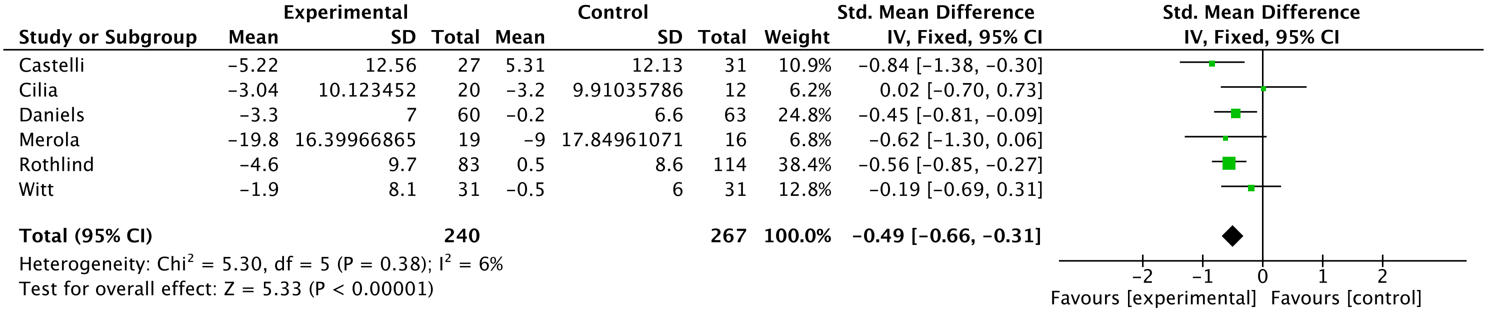

Figure S14. Forest plot of Phonemic fluency change STN DBS vs. Control

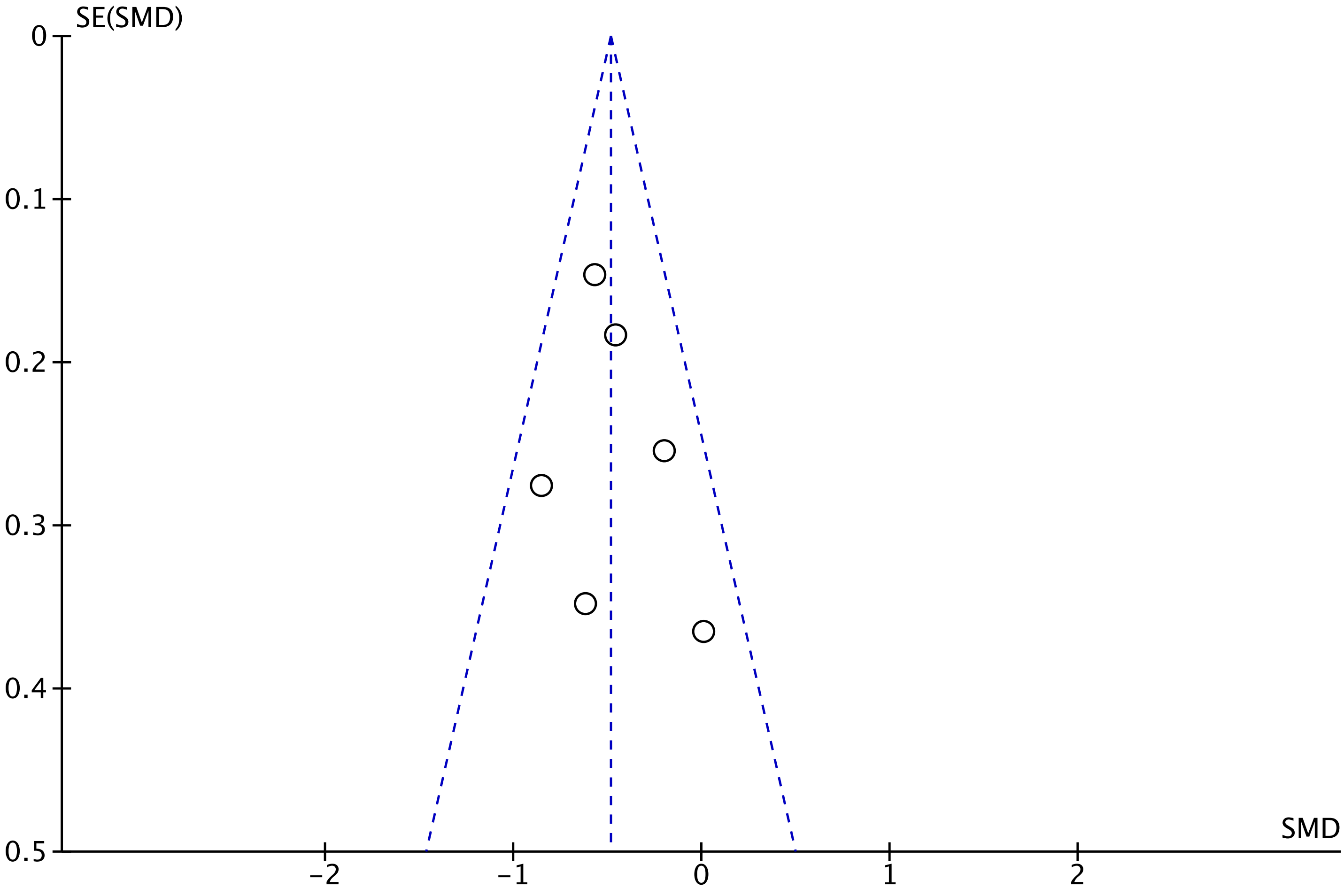

Figure S15. Funnel plot of Phonemic fluency change STN DBS vs. Control

	Experimental			Control				Std. Mean Difference
**Study or Subgroup**	**Mean**	**SD**	**Total**	**Mean**	**SD**	**Total**	**Weight**	**IV, Fixed, 95% CI**
Castelli	−1.43	3.34	27	0.07	7.94	31	21.0%	−0.24 [−0.75, 0.28]
Daniels	−5.6	9.7	60	−1.1	10.1	63	43.8%	−0.45 [−0.81, −0.09]
Williams	−3.2	4.94789	19	−2.6	5.1696	18	13.5%	−0.12 [−0.76, 0.53]
Witt	−6.1	11.6	31	0.3	10.3	31	21.7%	−0.58 [−1.08, −0.07]
**Total (95% CI)**			**137**			**143**	**100.0%**	**−0.39 [−0.63, −0.15]**

Heterogeneity: $Chi^2 = 1.65$, df = 3 (P = 0.65); $I^2 = 0\%$
Test for overall effect: Z = 3.21 (P = 0.001)

Std. Mean Difference
IV, Fixed, 95% CI

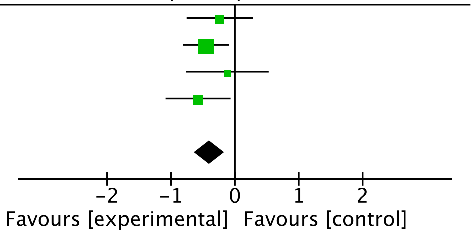

Figure S16. Forest plot of Semantic fluency change STN DBS vs. Control

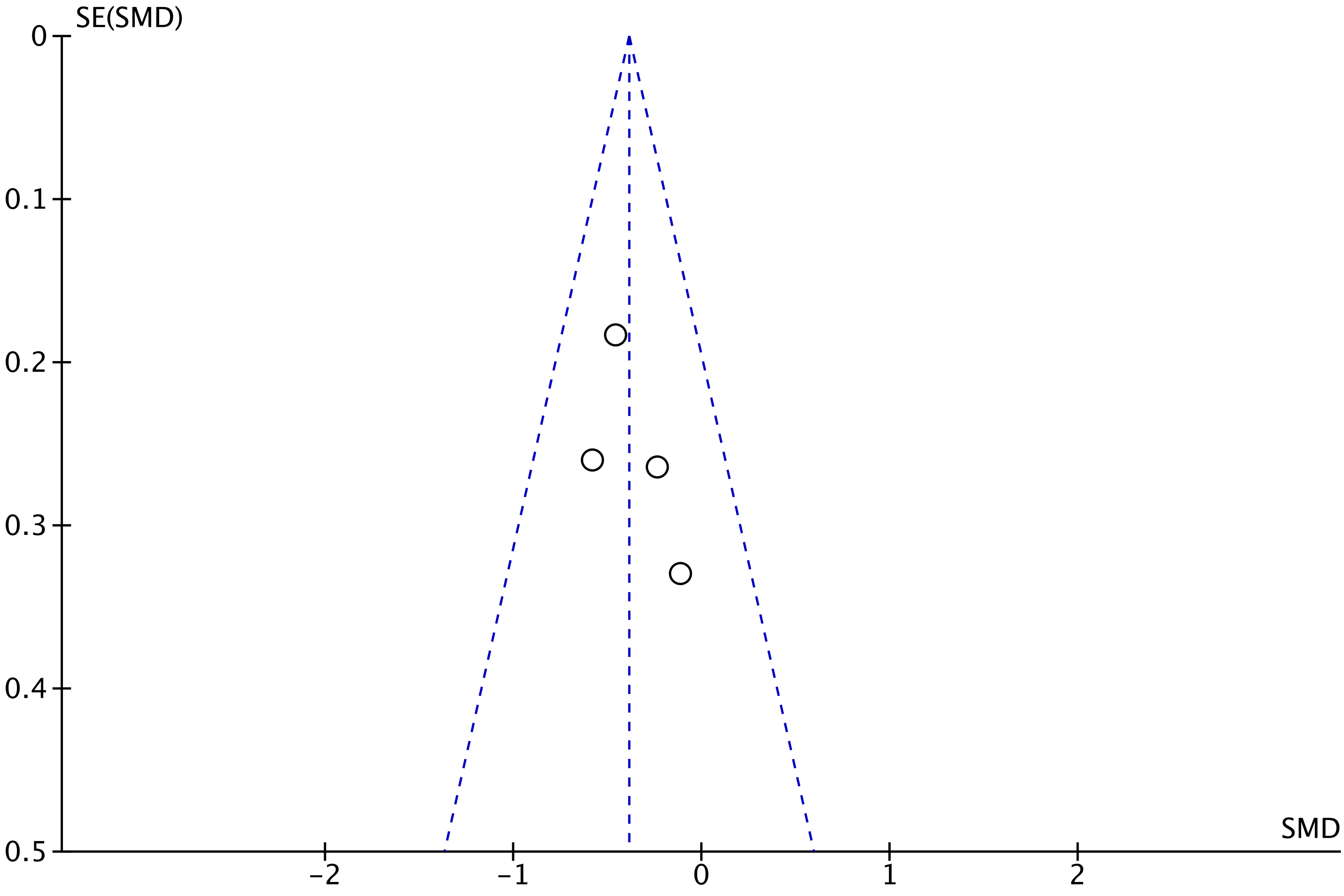

Figure S17. Funnel plot of Semantic fluency change STN DBS vs. Control

	Experimental			Control				Std. Mean Difference	Std. Mean Difference
**Study or Subgroup**	**Mean**	**SD**	**Total**	**Mean**	**SD**	**Total**	**Weight**	**IV, Fixed, 95% CI**	**IV, Fixed, 95% CI**
Castelli	−0.22	5.35	27	0.34	4.21	31	62.5%	−0.12 [−0.63, 0.40]	
Merola	−6.2	8.17537713	19	−4.2	9.69667969	16	37.5%	−0.22 [−0.89, 0.45]	
**Total (95% CI)**			**46**			**47**	**100.0%**	**−0.15 [−0.56, 0.25]**	

Heterogeneity: $Chi^2 = 0.06$, df = 1 (P = 0.81); $I^2 = 0\%$

Test for overall effect: Z = 0.74 (P = 0.46)

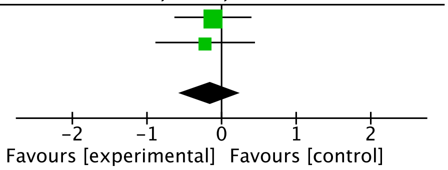

Figure S18. Forest plot of Reven Color Matrics change STN DBS vs. Control

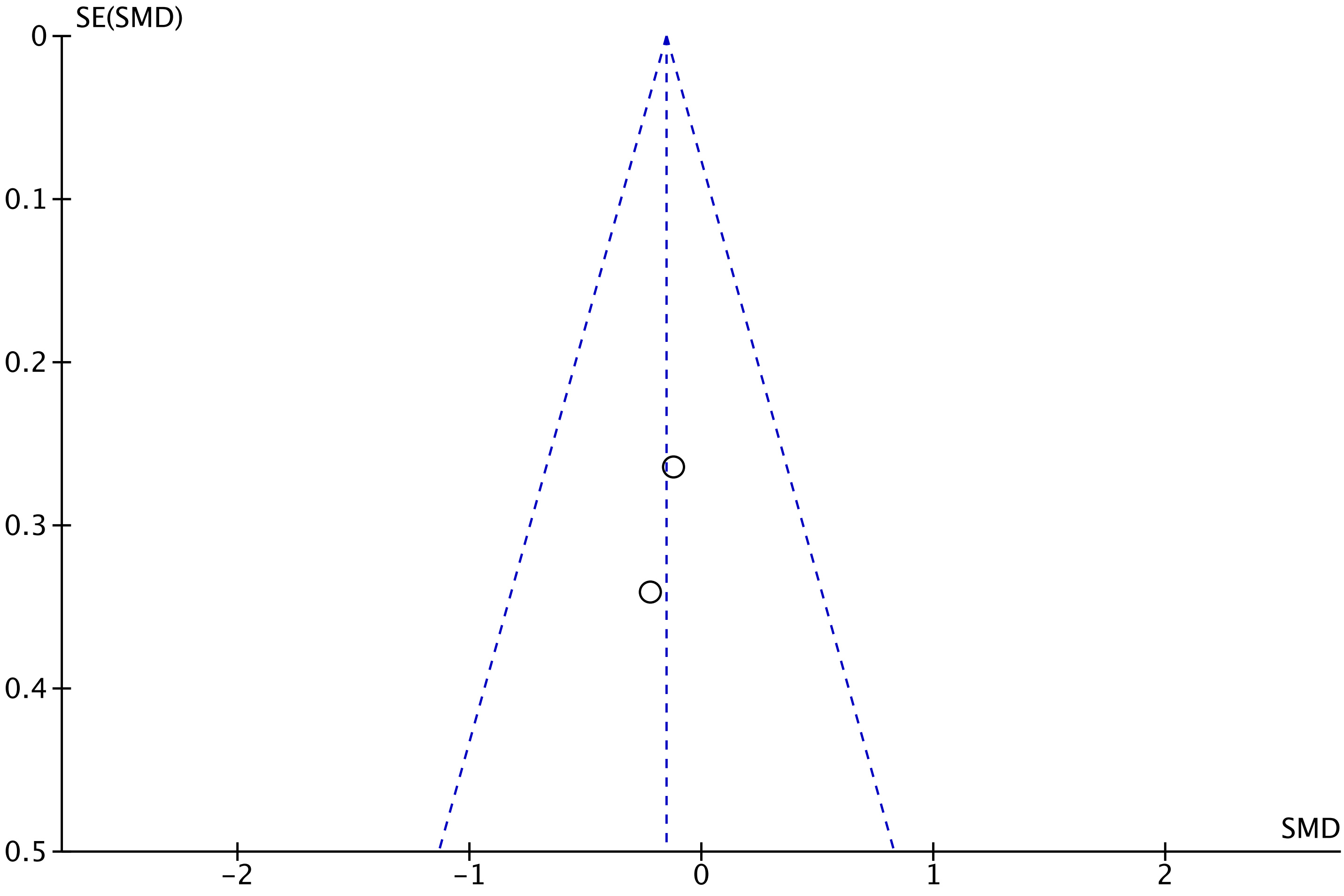

Figure S19. Funnel plot of Reven Color Matrics change STN DBS vs. Control

Study or Subgroup	Experimental Mean	Experimental SD	Experimental Total	Control Mean	Control SD	Control Total	Weight	Std. Mean Difference IV, Fixed, 95% CI
Daniels	−4.8	10.7	60	0.4	9.5	63	21.8%	−0.51 [−0.87, −0.15]
Rothlind	−2.8	6.3	78	0.5	8.4	109	32.5%	−0.43 [−0.73, −0.14]
Smeding	17	62.9	99	−13.8	32.1	36	18.8%	0.54 [0.16, 0.93]
Williams	−2.3	13.09854	19	−0.4	11.8252377	18	6.7%	−0.15 [−0.79, 0.50]
Witt	−12.3	51.1	31	0.3	18.3	31	11.2%	−0.32 [−0.83, 0.18]
York	−3.5	11.8742141	23	0	8.86887565	27	9.0%	−0.33 [−0.89, 0.23]
**Total (95% CI)**			**310**			**284**	**100.0%**	**−0.23 [−0.39, −0.06]**

Heterogeneity: $Chi^2 = 19.81$, df = 5 (P = 0.001); $I^2 = 75\%$

Test for overall effect: Z = 2.65 (P = 0.008)

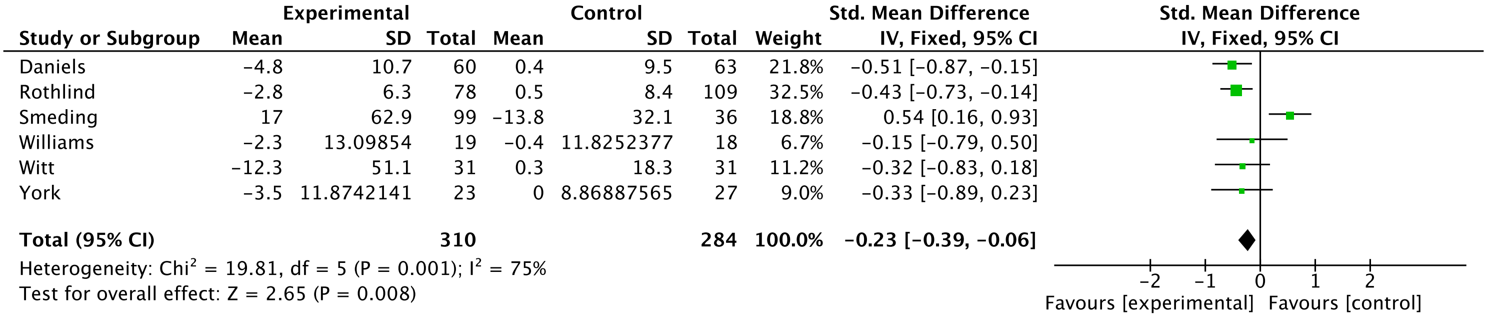

Figure S20. Forest plot of Stroop Color Word change STN DBS vs. Control

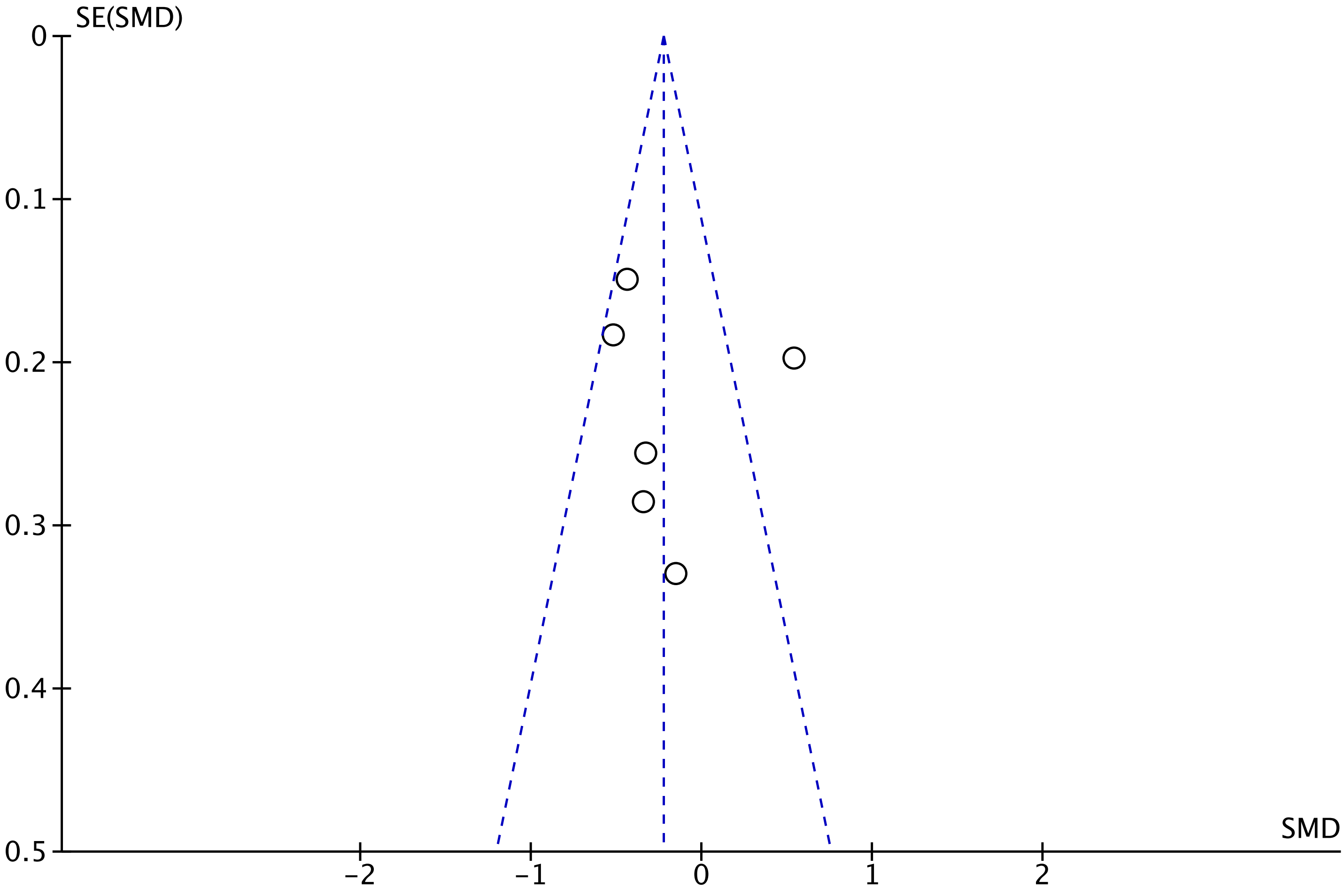

Figure S21. Funnel plot of Stroop Color Word change STN DBS vs. Control

Study or Subgroup	Experimental Mean	Experimental SD	Experimental Total	Control Mean	Control SD	Control Total	Weight	Std. Mean Difference IV, Fixed, 95% CI
Rothlind	0.4	2.4	81	0.4	1.9	114	68.7%	0.00 [−0.28, 0.28]
Williams	1.2	6.5028232	19	0.2	2.43349321	18	13.3%	0.20 [−0.45, 0.84]
York	0.1	7.75308893	23	0.2	2.40037481	27	18.0%	−0.02 [−0.57, 0.54]
**Total (95% CI)**			**123**			**159**	**100.0%**	**0.02 [−0.21, 0.26]**

Heterogeneity: $Chi^2 = 0.32$, df = 2 (P = 0.85); $I^2 = 0\%$
Test for overall effect: Z = 0.19 (P = 0.85)

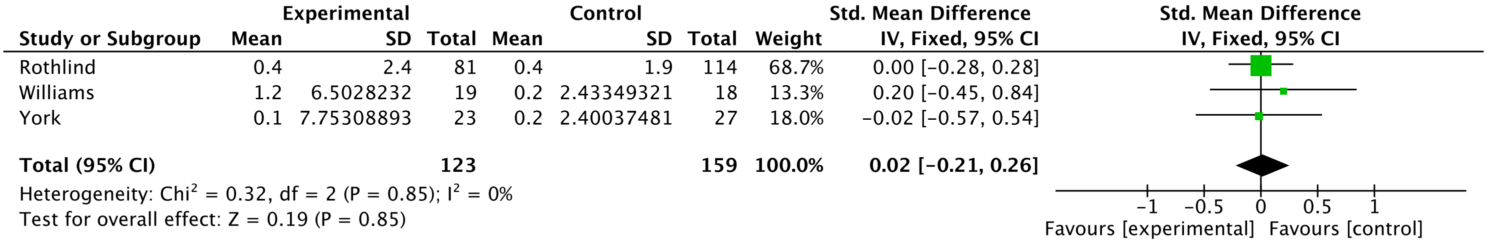

Figure S22. Forest plot of Boston Naming change STN DBS vs. Control

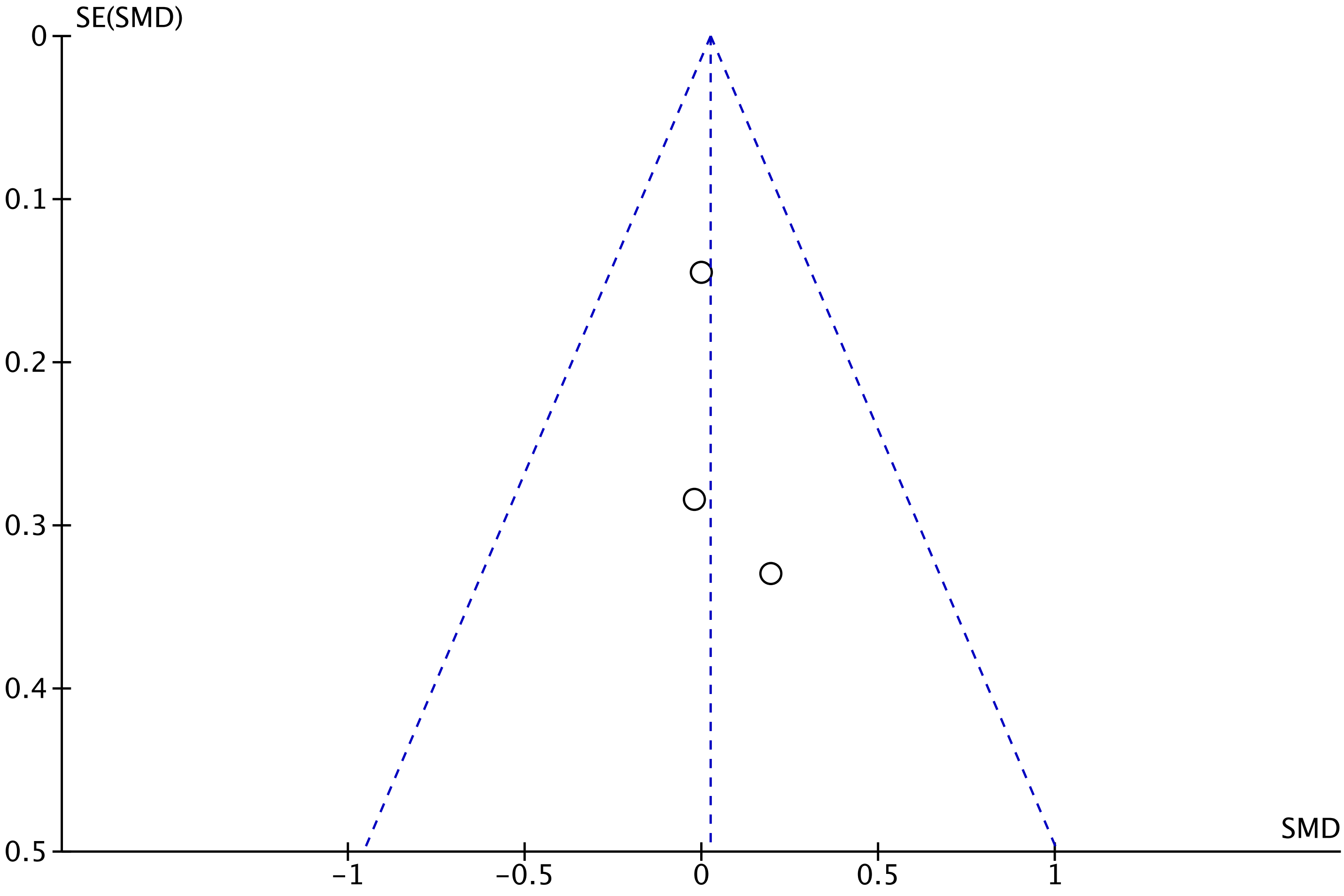

Figure S23. Funnel plot of Boston Naming change STN DBS vs. Control

Study or Subgroup	Experimental Mean	Experimental SD	Experimental Total	Control Mean	Control SD	Control Total	Weight	Std. Mean Difference IV, Fixed, 95% CI
Rothlind	−0.7	24.2	84	1	18	115	50.4%	−0.08 [−0.36, 0.20]
Smeding	0.3	19	99	−2.6	15.6	36	27.3%	0.16 [−0.22, 0.54]
Williams	8.1	22.03698636	19	13.9	48.16208934	18	9.6%	−0.15 [−0.80, 0.49]
York	7.4	26.87169683	23	−0.7	27.64709091	27	12.7%	0.29 [−0.27, 0.85]
**Total (95% CI)**			**225**			**196**	**100.0%**	**0.03 [−0.17, 0.22]**

Heterogeneity: $Chi^2 = 2.19$, df = 3 (P = 0.53); $I^2 = 0\%$
Test for overall effect: Z = 0.25 (P = 0.81)

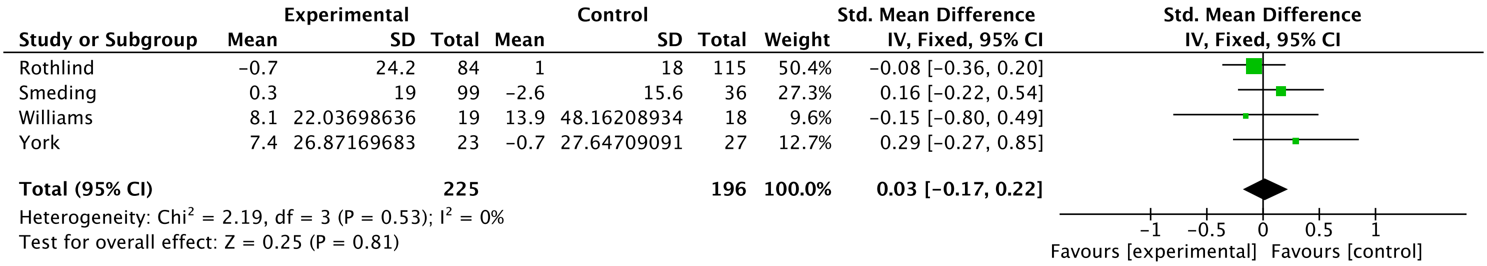

Figure S24. Forest plot of Trial Making a change STN DBS vs. Control

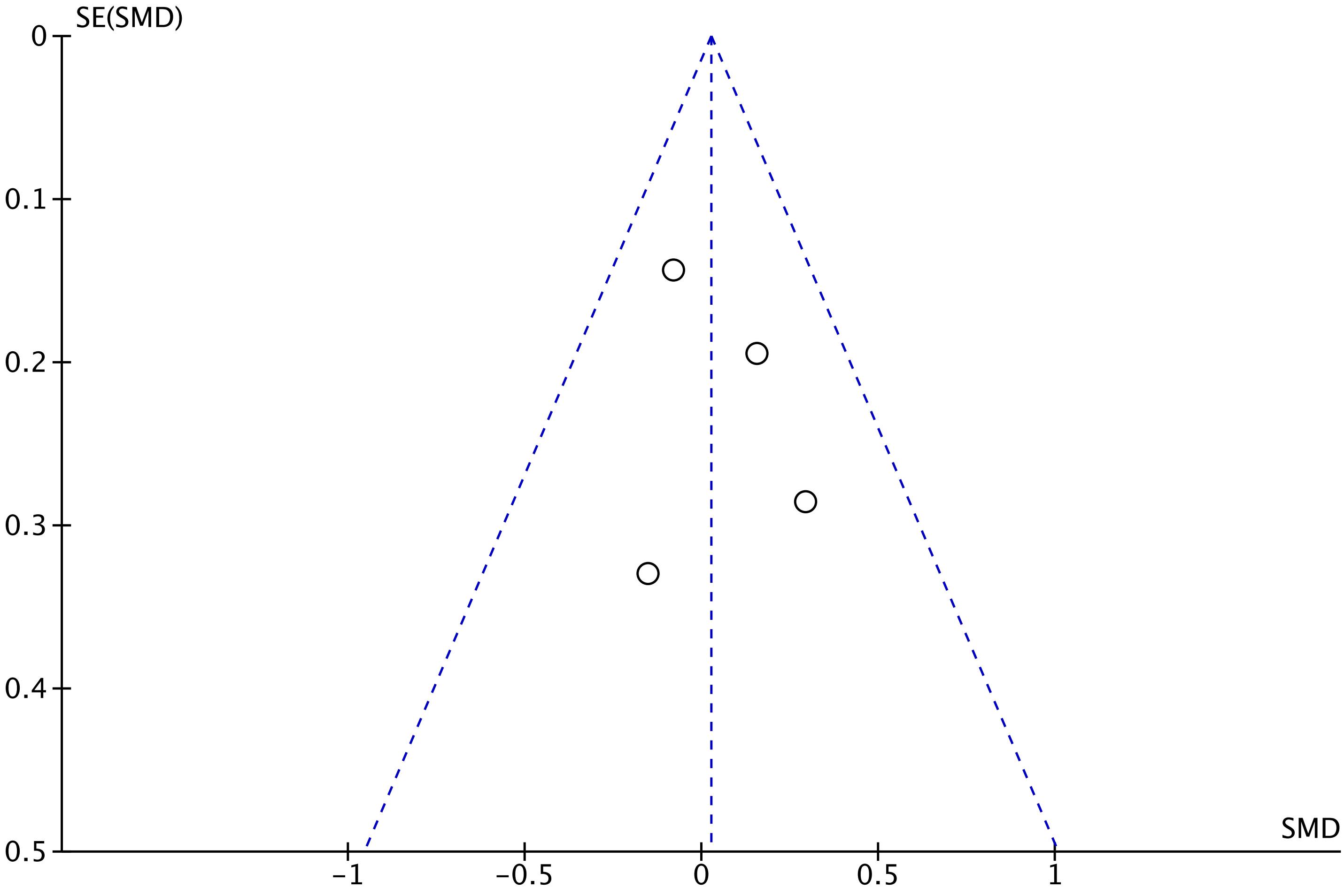

Figure S25. Funnel plot of Trial Making a change STN DBS vs. Control

	Experimental			Control				Std. Mean Difference
Study or Subgroup	Mean	SD	Total	Mean	SD	Total	Weight	IV, Fixed, 95% CI
Castelli	12.7	193.55	27	−5	114.14	31	13.2%	0.11 [−0.40, 0.63]
Merola	−1,985.4	157.8703372	19	26.5	211.0738267	16	0.5%	−10.69 [−13.42, −7.95]
Rothlind	9.7	50.8	81	9.1	60.9	112	43.1%	0.01 [−0.28, 0.30]
Smeding	19.3	91.8	99	8.7	42.4	36	24.1%	0.13 [−0.25, 0.51]
Williams	40.9	92.32207955	19	−24.9	75.56682451	18	7.8%	0.76 [0.09, 1.43]
York	25.1	84.27817013	23	10.1	61.25765378	27	11.3%	0.20 [−0.35, 0.76]
**Total (95% CI)**			**268**			**240**	**100.0%**	**0.08 [−0.10, 0.27]**

Heterogeneity: $Chi^2 = 63.79$, df = 5 ($P < 0.00001$); $I^2 = 92\%$
Test for overall effect: Z = 0.87 (P = 0.39)

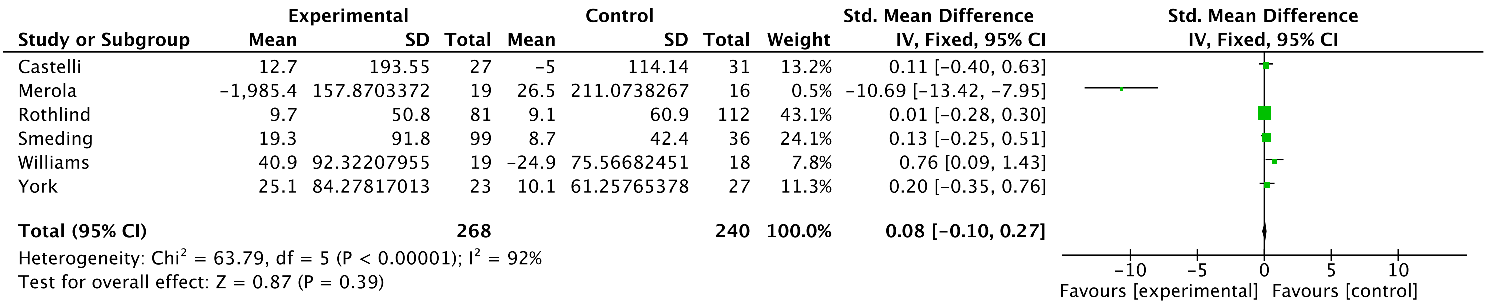

Figure S26. Forest plot of Trial Making b change STN DBS vs. Control

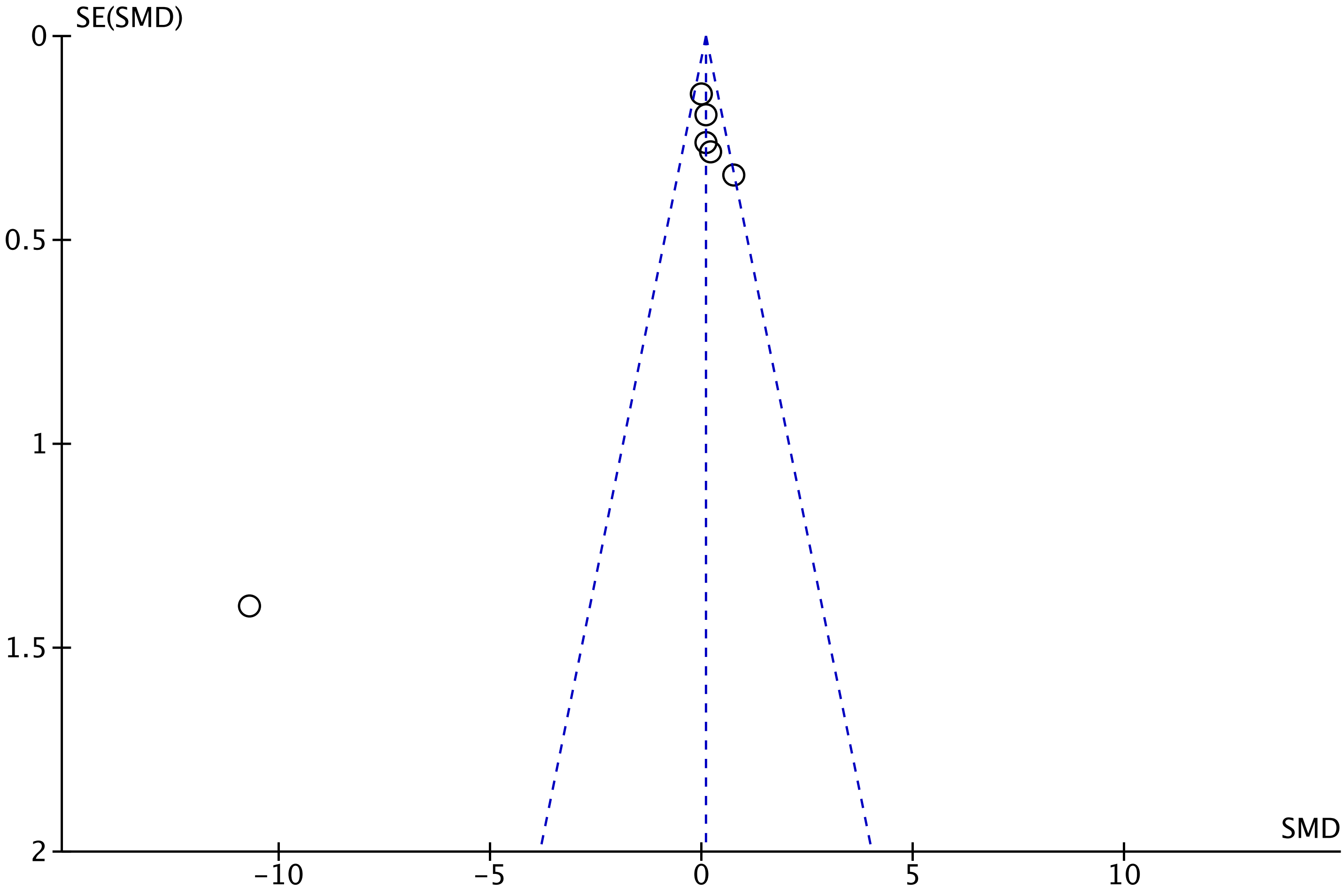

Figure S27. Funnel plot of Trial Making b change STN DBS vs. Control

Study or Subgroup	Experimental Mean	Experimental SD	Experimental Total	Control Mean	Control SD	Control Total	Weight	Mean Difference IV, Fixed, 95% CI
Castelli	−0.39	2.66	27	0.47	3.49	31	69.6%	−0.86 [−2.45, 0.73]
Merola	−0.6	3.48239328	19	−0.3	3.70532767	16	30.4%	−0.30 [−2.70, 2.10]
**Total (95% CI)**			**46**			**47**	**100.0%**	**−0.69 [−2.01, 0.63]**

Heterogeneity: $Chi^2 = 0.15$, df = 1 (P = 0.70); $I^2 = 0\%$
Test for overall effect: Z = 1.02 (P = 0.31)

Mean Difference IV, Fixed, 95% CI

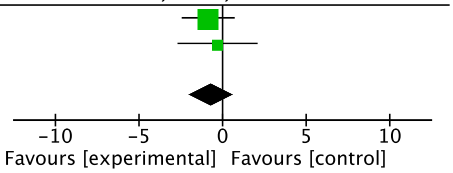

Figure S28. Forest plot of Paired associate learning change STN DBS vs. Control

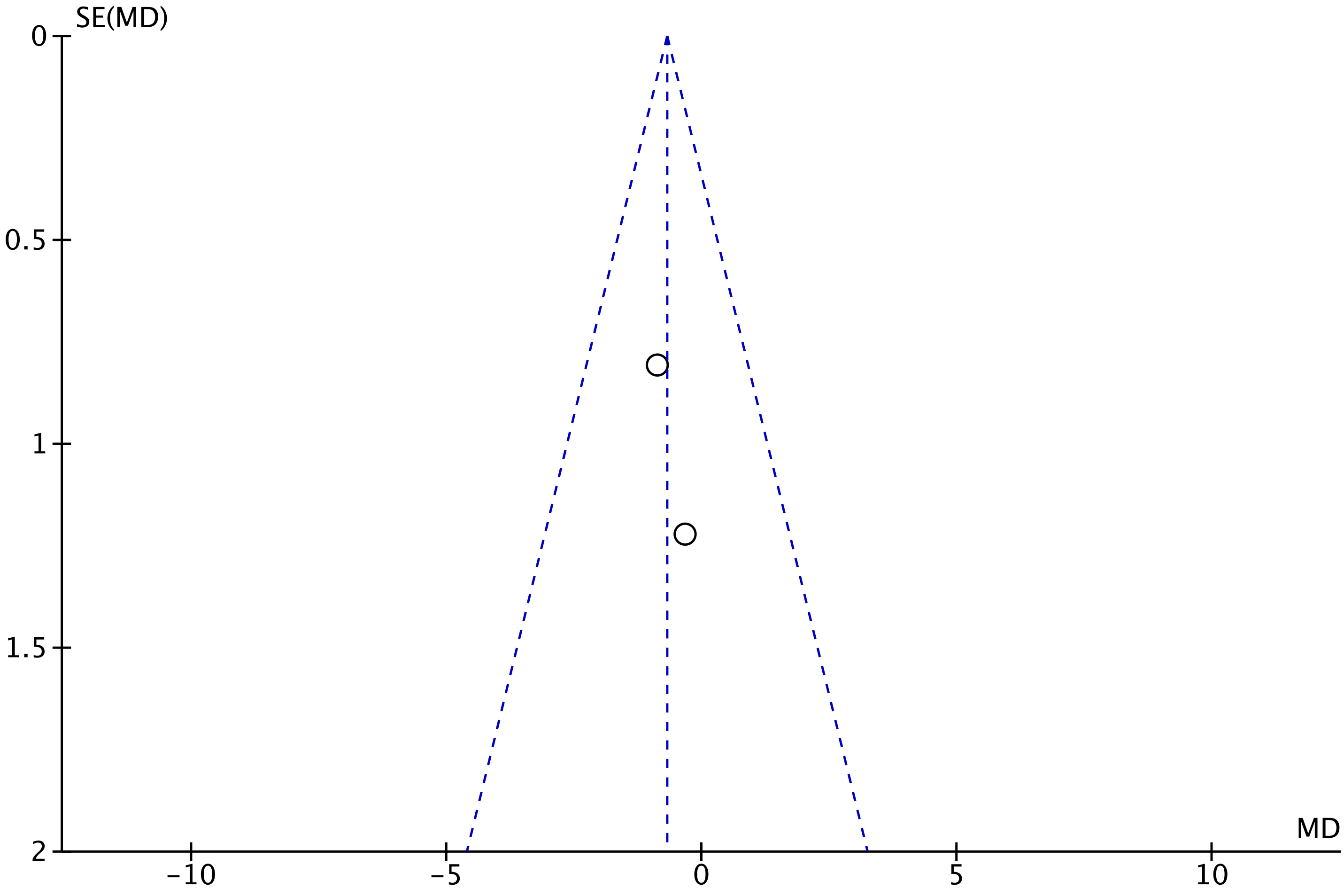

Figure S29. Funnel plot of Paired associate learning change STN DBS vs. Control

Study or Subgroup	Experimental Mean	Experimental SD	Experimental Total	Control Mean	Control SD	Control Total	Weight	Mean Difference IV, Fixed, 95% CI
Daniels	−2	8.2	60	−0.48	9.49	63	40.8%	−1.52 [−4.65, 1.61]
Smeding	−0.8	9.7	99	1.6	7	36	45.0%	−2.40 [−5.38, 0.58]
Williams	−4.6	11.57101604	19	−3.1	14.86701423	18	5.4%	−1.50 [−10.12, 7.12]
York	−3.3	12.33938782	23	−0.1	11.77229489	27	8.8%	−3.20 [−9.92, 3.52]
**Total (95% CI)**			**201**			**144**	**100.0%**	**−2.06 [−4.06, −0.06]**

Heterogeneity: $Chi^2 = 0.29$, df = 3 (P = 0.96); $I^2 = 0\%$

Test for overall effect: Z = 2.02 (P = 0.04)

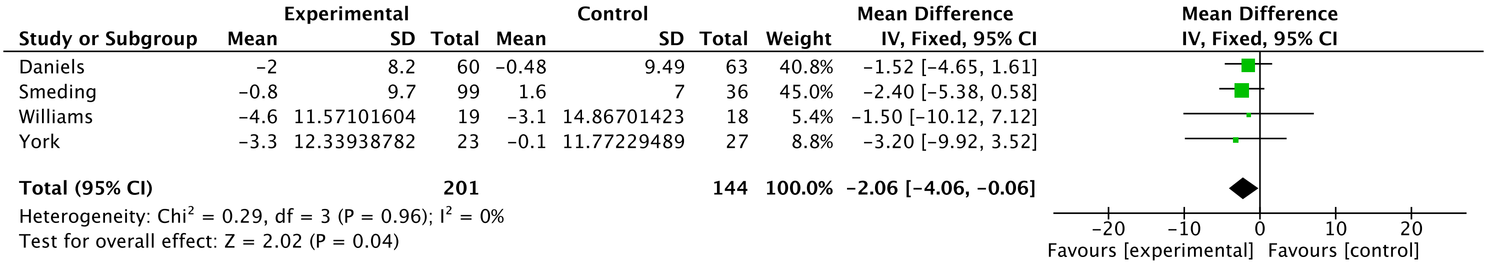

Figure S30. Forest plot of RAVLT-total change STN DBS vs. Control

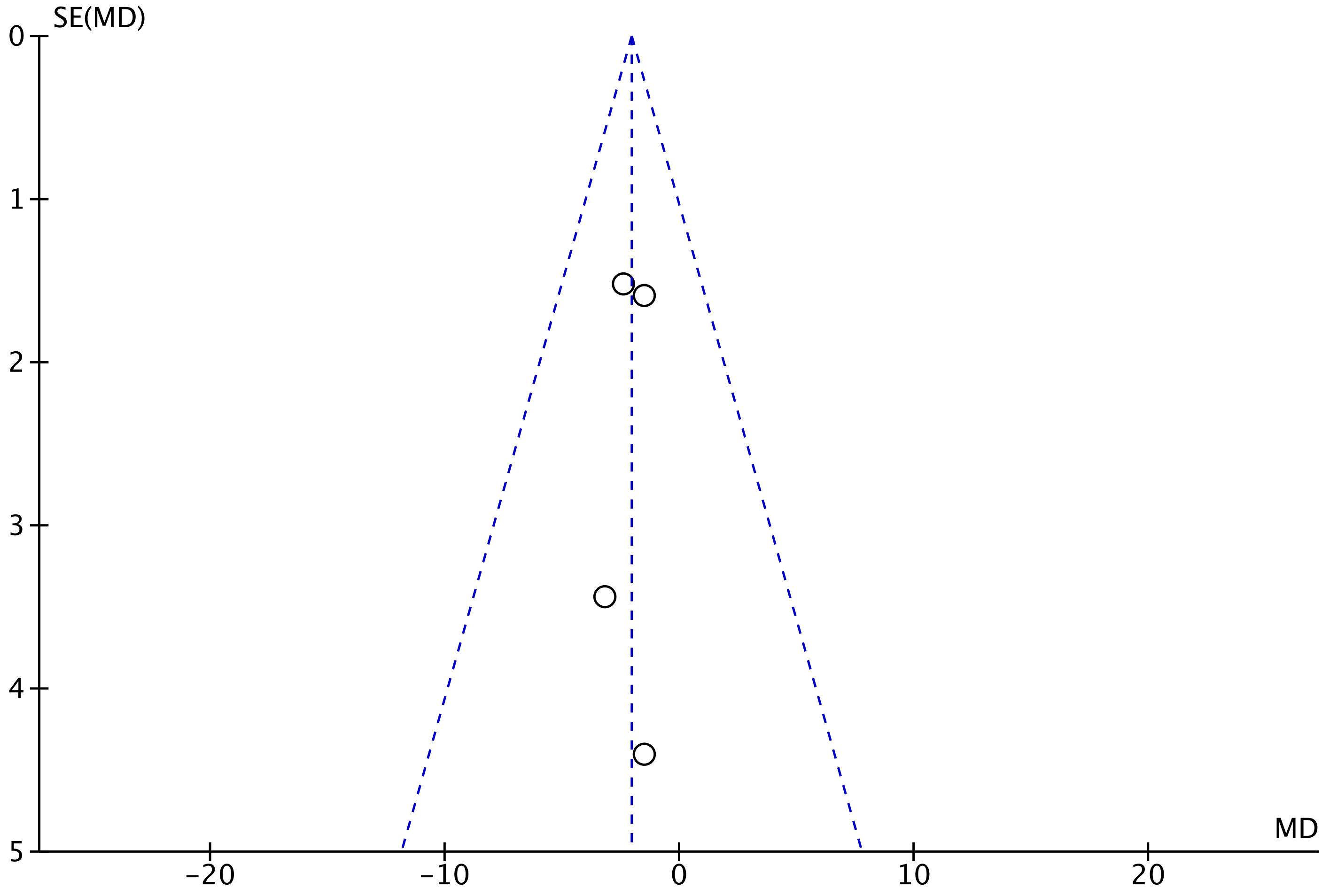

Figure S31. Funnel plot of RAVLT-total change STN DBS vs. Control

	Experimental			Control				Mean Difference
**Study or Subgroup**	**Mean**	**SD**	**Total**	**Mean**	**SD**	**Total**	**Weight**	**IV, Fixed, 95% CI**
Smeding	−0.8	3.2	99	0.5	2.2	36	74.6%	−1.30 [−2.26, −0.34]
Williams	−1.9	4.06582508	19	−0.28	4.09224604	18	9.9%	−1.62 [−4.25, 1.01]
York	−1.6	3.84620666	23	0.2	3.68529422	27	15.5%	−1.80 [−3.90, 0.30]
**Total (95% CI)**			**141**			**81**	**100.0%**	**−1.41 [−2.23, −0.58]**

Heterogeneity: $Chi^2 = 0.21$, df = 2 (P = 0.90); $I^2 = 0\%$
Test for overall effect: Z = 3.34 (P = 0.0008)

Mean Difference
IV, Fixed, 95% CI

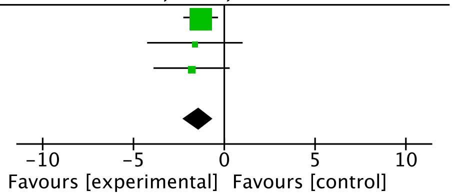

Figure S32. Forest plot of RAVLT-delayed recall change STN DBS vs. Control

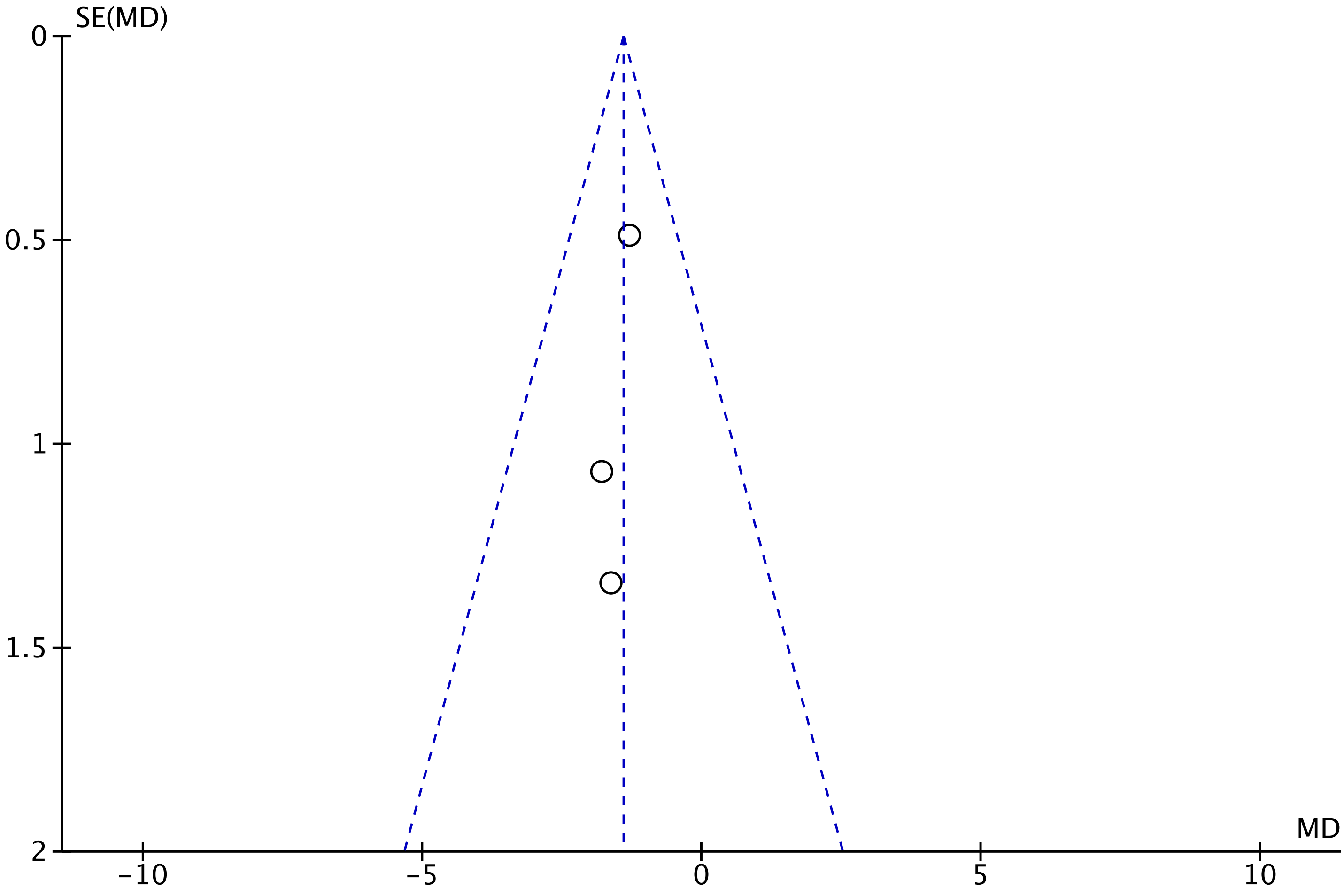

Figure S33. Funnel plot of RAVLT-delayed recall change STN DBS vs. Control
